# Guessing less and better: improved attacks on GIFT-64

**DOI:** 10.1007/s10623-024-01527-2

**Published:** 2024-12-20

**Authors:** Federico Canale, María Naya-Plasencia

**Affiliations:** 1 Horst Görtz Institute for IT Security, Ruhr University Bochum, Bochum, Germany; 2 Inria, Paris, France

**Keywords:** Key-recovery, Differential cryptanalysis, Parallel guessing, List merging, Generic framework, GIFT-64, 94A60

## Abstract

GIFT-64 is a block cipher that has received a lot of attention from the community since its proposal in 2017. The attack on the highest number of rounds is a differential related-key attack on 26 rounds. We studied this attack, in particular with respect to some recent generic frameworks for improving key recovery, and we realised that this framework, combined with an efficient parallel key guessing of interesting subsets of the key and a consequent list merging applied to the partial solutions, can improve the complexity of the attack. We propose two different trade-offs, as a result of the improved key-recovery. We believe that the techniques are quite generic and that it is possible to apply them to improve other differential attacks.

## Introduction

Among the most successful classes of attacks against modern symmetric primitives there are undoubtedly the statistical attacks. In a nutshell, in each of these attacks a certain property exhibited in a statistically significant way by the encryption algorithm is used to distinguish it from a random function. This distinguishing property is then used to reduce the space of possible keys to the ones that pass the statistical test. This reduced list of key candidates is usually determined by a key enumeration algorithm, usually referred to as key guessing.

A special interest in introducing new ideas for improving the key-recovery phase in statistical attacks has been carried out recently [4, 5, 16]. In particular, a generic framework for improving key-recovery attacks that exploits the Sbox properties to reduce the number of bits needed to guess on average was presented in [5]. This framework can be applied to several cryptanalysis families, including differential cryptanalysis.

The block cipher GIFT [1], proposed in 2017 has received much attention from the community, as the numerous cryptanalysis results ([7, 8, 11, 14, 15] among many) can show. In [13] the currently best known attack on reduced round versions of the 64-bit version of GIFT is proposed, a related-key differential attack on 26-rounds that is presented in Table 1.

In this paper we consider the generic framework for improving key guessing from [5] and we combine it with a clever parallel guess of the key bits in the previous best differential attacks on GIFT [13] in order to improve their complexity and become the best known attacks on GIFT. Besides showing a new application of the techniques from [5] and an improved attack on the cipher that gains a time factor of about $2^{10}$ (while the previous attack had a complexity close to exhaustive search), we believe our results propose a parallel guessing method that can be generalized and applied in many other differential attacks. Furthermore, we show how the improvement in enumerating the possible key guesses can lead to an attack that reduces significantly the necessary memory. In addition, we have implemented the techniques presented in this paper on a toy-cipher, which has allowed us to verify our theoretical predictions. The results are summarised in Table 1. In a simultaneous and independent recent work, published at Eurocrypt 2022 [9], an improved attack with respect to [13] was proposed, but this work uses different techniques which lead to worse complexities than the improved one we propose here.

The paper is organized as follows: Sect. 2 introduces some notations and preliminaries about differential attacks, GIFT and key guessing techniques from [5] and Sect. 3 summarises the main ideas of the attack. The previous 26-round related-key attack on GIFT-64 [13] and its complexity is discussed in Sect. 4, while our new improved attacks are introduced in Sect. 5. Section 6 concludes the paper. The implementations on a GIFT-like toy-cipher that we have done in order to verify the correctness of our techniques are described in Appendix E.^1^

**Table 1:** Summary of results on GIFT-64

Rounds	Source	Attack type	Time	Data	Memory
24	[14]	Rectangle (RK)	$2^{91.58}$	$2^{60}$	$2^{60.32}$
25	[10]	Rectangle (RK)	$2^{120.92}$	$2^{63.78}$	$2^{64.10}$
26	[13]	Differential (RK)	$2^{123.23}$	$2^{60.96}$	$2^{102.86}$
26	[9]	Rectangle (RK)	$2^{122.78}$	$2^{63.78}$	$2^{63.78}$
26	This paper	Differential (RK)	$2^{113.03}$	$2^{61.96}$	$2^{95.15}$
26	This paper	Differential (RK)	$$2^{120.44}$$	$$ 2^{60.96}$$	$$2^{25.22}$$

## Preliminaries

In this section we introduce some of the notations that will be used throughout the paper, as providing an overview of GIFT, differential cryptanalysis and the techniques introduced in [5] that we are going to use.

### Description of GIFT-64

GIFT-64 is a block cipher first introduced in [1] of block size 64 and key length 128. The 64-bit state consists of 16 4-bit nibbles which will be denoted by $b_{0} \ldots b_{63} = x_{0}\Vert \ldots \Vert x_{15}$. Following the well-known SPN design, each round of GIFT-64 consists of three steps: the application in parallel of 16 4-bit Sboxes, a bit permutation, and the addition of a 32-bit subkey. We here present the specifications of GIFT according to the representation used in the original paper of the attack we improve upon [13], where the bit ordering is mirrored with respect to the original paper [1] (where the least significant bit is the rightmost one, etc.).

#### The GIFT Sbox

The GIFT SBox *GS* is given below.

**Table:** 

$\pmb {x }$	0	1	2	3	4	5	6	7	8	9	a	b	c	d	e	f
$\pmb {GS(x)}$	8	4	6	a	2	d	c	1	5	b	f	0	3	e	9	7

#### Bit permutation

As a linear layer, GIFT uses the permutation

$$\begin{aligned} P_{64}(\pi (i))&= \pi \Bigg (4 \left\lfloor \frac{\pi (i)}{16} \right\rfloor + 16 \left( \left( 3 \left\lfloor \frac{\pi (i) \ \textrm{mod} \ 16}{4}\right\rfloor +\left( \pi (i) \ \textrm{mod} \ 4\right) \right) \textrm{mod} \ 4\right) \\&\quad + \left( \pi (i) \ \textrm{mod} \ 4 \right) \Bigg ). \end{aligned}$$

where $\pi$ is the permutation that mirrors the bits, that is $\pi (i)= (63-i) \ \textrm{mod} \ 64$.

GIFT-64 uses 32-bit round subkeys which are XORed to the bit positions of the state of the form $b_{4i+2},b_{4i+3}, \ i=0,\ldots ,15$ (that is, the two rightmost bits of each Sbox before the non-linear layer).

#### Key addition and key schedule

The round keys of GIFT-64 are 32 bits, as for each nibble of the state only the two most significant bits are mixed with the round key.

The key state is initialized by the master key *K* and split into eight 16-bit substates, $K=k_0, \ldots , k_7$. A round key is extracted from a two 16-bit words of the key state extracted as $RK = U||V$:

$$\begin{aligned} U \leftarrow k_6, V \leftarrow k_7. \end{aligned}$$

The key state is then updated as follows: $k_0 \Vert k_1 \Vert \ldots \Vert k_7 \leftarrow k_6 \lll 2 \Vert k_7 \lll 12 \Vert \ldots \Vert k_4 \Vert k_5$, where $\lll i$ is an *i* bits left rotation within a 16-bit word. Finally, round constants generated by a 6-bit LFSR are added to the state. For further details we refer to [1].

### Differential cryptanalysis

Differential cryptanalysis, introduced in [2], is a family of statistical attack that aims at distinguishing a non-ideal permutation by studying the propagation of differences. Formally, a related key differential distinguisher on $E_1: \mathbb {F}_2^n \times \mathbb {F}_2^m \rightarrow \mathbb {F}_2^n$ is a triplet $(\delta ,\Delta , \gamma )\in \mathbb {F}_2^{2n+m}$ such that

$$\begin{aligned} \text {Pr}_{x \in \mathbb {F}_2^n}\{E_1(x,k) \oplus E_1(x\oplus \delta , k\oplus \gamma )=\Delta \} =p > 2^{-n} \end{aligned}$$

for any fixed key *k*. This means that for every $p^{-1}$ pairs having the desired input difference $\delta$ and encrypted with the same pair of keys related by the difference $\gamma$, we expect that one will yield the output difference $\Delta$; otherwise, this would happen randomly, *i.e.* once every $2^n$ pairs. Since this class of attacks depend on keeping track of how many pairs result in a certain output difference $\Delta$, we refer to such pairs as *good pairs*.

More generally, let $I, O\subset \mathbb {F}_2^n$ and $f:\mathbb {F}_2^n \rightarrow \mathbb {F}_2^n$. We say that $(x, \tilde{x})$ is a good pair for a differential transition $I {\mathop {\rightarrow }\limits ^{f}} O$ if $(x, \tilde{x}) \in \delta$ and $f(x)\oplus f(\tilde{x})\in O$ for all $\delta \in I$.

A key recovery attack using such a related key distinguisher can be mounted on the $r^\prime +r$ rounds cipher $E_1 \circ E_0$ (where $E_0$ is $r^\prime$ rounds) by guessing the necessary key bits needed to compute, for each key guess, many pairs $(m, \widetilde{m})$ such that $E_0(m, k)\oplus E_0(\widetilde{m}, k\oplus \gamma )=\delta$ (usually $p^{-1}$ such pairs, if *p* is the probability of the distinguisher). In fact, if this is the case, we would expect that for the right key-guess one in every $p^{-1}$ pairs would yield the expected output difference, whereas for a wrong key-guess the encryption algorithm would behave like a random permutation, so that the expected output difference would only appear once every $2^n$ pairs. Clearly, the same can be done by appending $r^{\prime \prime }$ rounds of key guessing $E_2$ after $E_1\circ E_0$.

We will consider the notion of good triplets (formed by a pair of data and an associated subkey for which the pair is a good pair, *i.e.* satisfies the differential). We denote the maximum number of triplets at any point of the attack as *T*. The term associated to this step is likely to be the bottleneck in the estimation of the time complexity. If, for instance, we consider $\tau _1$ as the average complexity (in terms of encryptions) needed to establish whether a single triplet is good or not, then

$$\begin{aligned} T_1 \approx \tau _1 \cdot T \end{aligned}$$

is the term we will need to pay in the complexity for retrieving all the good triples. After this step, we want to retrieve the secret key out of the candidates given by the triplets. It will be possible to filter a certain number of master subkey candidates, according to statistic $\Sigma _k$ that is usually the number of times a triplet vote for a specific value *k*, that is if $\theta$ is the threshold that we set for a possible subkey value, then we want to estimate the so called false flag probability

$$\begin{aligned} \beta =\Pr (\Sigma _k \ge \theta ) \end{aligned}$$

so that we expect $2^{128}\beta$ keys will pass the first step. Therefore, we expect $T_2=2^{128}\beta (1-2^{-64})$ encryptions to finally recover the entire master key.

Finally, the overall complexity of the attack is

$$\begin{aligned} T=T_0+T_1+T_2 \end{aligned}$$

where $T_0$ is the number of encryptions necessary to prepare the plaintext ciphertext pairs in case we use, for example, structured data.

To estimate the probability of false flag and the probability of success of this procedure, we refer to [3].

#### Notations

When analysing a single Sbox *GS* (resp. $GS^{-1}$), we will use the convention of naming the input bits as $x_3, \ldots , x_0$ and the output bits as $y_3, \ldots , y_0$. The key material XORed to the input bits $x_3, x_2$ will be called $k=(k_1, k_0)$. We will sometimes write, with abuse of notation, $x\oplus k$ meaning $x\oplus (k_1, k_0,0,0)$. When necessary, the bits of key that are XORed to the output bits $y_0, y_1$ in the later (resp. earlier) round will be called $\kappa _1, \kappa _0$. Notice that the GIFT permutation modulo 4 is the identity map, meaning that least significant bits are sent to least significant bits, and so on.

When talking about the intermediate states of the encryption algorithm, we will use the same notations of [13] (*i.e.* $X^{i}, Y^{i}$ are the states before and after the Sbox layer of round *i*). We then call $\Delta ^i$ the differences required by the distinguisher in round *i*, and $RK^i$ the *i*-th round key.

For any vector $V=(v_{n}, \ldots , v_{0})$, we indicate by $V[i_l, \ldots , i_0]$ the vector $(v_{i_l},\ldots , v_{i_0})$ or by $V[\alpha ]$, the bit $\langle \alpha , V \rangle =v_{i_l}\oplus \ldots \oplus v_{i_0}$ if $\alpha _{j}=1$ if and only if $j\in \{i_0, \ldots , i_l\}$. Let $S\subset \mathbb {F}_2^4$, we write that $S=A_3 A_2 A_1 A_0$ where, for each $j=0, \ldots , 3$, $A_j=i$ if $x_j=i$ for all $x\in S$ ($i\in \{0,1\}$), otherwise $A_j=X$; in other words, $A_j=X$ whenever the *j*-th bit of the set of differences is not set. For example, $XXXX=\mathbb {F}_2^4$ and $X000=\{\texttt {0},\texttt {8}\}$. Finally, let $I, O\subset \mathbb {F}_2^n$ and $f: \mathbb {F}_2^n \rightarrow \mathbb {F}_2^n$. We indicate by $\nabla _{I \rightarrow O}$ the set of possible values that an element of a good pair can take, that is

$$\begin{aligned} \{x \in \mathbb {F}_2^n: f(x)\oplus f(x\oplus \delta )\in O \hbox { for some}\ \delta \in I\}. \end{aligned}$$

### Tree-based key-recovery techniques and key absorption

We remind briefly the techniques introduced in [4] that are relevant to our attack.

#### Tree-based guessing

Suppose we want to determine $f(x\oplus k)$, where $f:\mathbb {F}_2^n\rightarrow \mathbb {F}_2^{m}$ is a partial *m*-bit output of an Sbox, for many possible *k*. This is often the case when guessing over multiple rounds, where the output bit of an Sbox is needed to determine a certain property on a later round. Instead of naively guessing the full *n*-bit word of *k*, the authors of [5] introduce a dynamic key-guessing technique from the observation that *f* can be described as a binary-decision tree, and an efficient guessing strategy can be carried out according to such trees. We will not go into the details of this but we provide the trees used in this paper for the relevant output bits of both *GS* and $GS^{-1}$ in Appendix A.

As an example, if *f* is the function that computes the output bit $y_0$ of *GS*, we will use ta tree based guessing strategy to determine it such that we need to guess, on average and depending on the concrete values, less that the two key bits. Here we consider $z=x\oplus (k_1, k_0,0,0)=(x_3\oplus k_1, x_2\oplus k_0, x_1, x_0)$ to be the input of *f*, with $k_0, k_1$ unknown:

1. if $\langle x, \texttt {1} \rangle = x_0=0$, then $y_0=\langle z, \texttt {8} \rangle =x_3\oplus k_1$. Therefore, only $k_1$ is necessary to determine $y_0$.
2. if $$\langle x, \texttt {1} \rangle = x_0=1$$, then
  if $\langle x, \texttt {2} \rangle = x_1=0$, $y_0= \langle z, \texttt {c} \rangle =x_3 \oplus x_2 \oplus k_1\oplus k_0$ and only $k_0\oplus k_1$ needs to be guessed additionally.
  if $\langle x, \texttt {2} \rangle = x_1=1$, $y_0= \langle z, \texttt {4} \rangle = x_2 \oplus k_0$ and only bit $k_0$ needs to be guessed additionally.

This strategy allows to compute $y_3$ with an average of one key bit guessed instead of 2 for every Sbox where it is needed.

#### Key absorption

We describe the key absorption technique in the case of GIFT for simplicity. Let us consider the following general case for GIFT, where we need to determine an output bit *j* of *GS* for some round *i*, $Y^{i}[j]$, in order to determine the corresponding bit of the next round $X^{i+1}[P(j)]=Y^{i}[j]\oplus RK^{i}[m]$ for some round key bit *m*. The naive guessing strategy would be to guess the two bits necessary to determine $Y^{i}[j]$ and then the bit $RK^{i}[m]$. This is however not necessary using the key absorption technique.

In fact, let us write $Y^{i}[j]$ as a linear relation on a bit of the input state $X^i$ that is we find $\alpha , \beta$ such that

1 $$\begin{aligned} Y^{i}[j]=Y^{i-1}[\alpha ]\oplus RK^{i-1}[\beta ]\oplus c \end{aligned}$$

for some $c\in \{0,1\}$. This relation can be found, for instance, among the ones given by the appropriate trees. Then

$$\begin{aligned} X^{i+1}[P(j)]=Y^{i}[j]\oplus RK^{i}[m]= Y^{i-1}[\alpha ]\oplus RK^{i-1}[\beta ]\oplus c\oplus RK^{i}[m]. \end{aligned}$$

Therefore, in order to determine $X^{i+1}[P(j)]$ we can simply guess $RK^{i-1}[\beta ]\oplus c\oplus RK^{i}[m]$ and the round key bit that we guessed to find the relation of Eq. (1). We remark that in the case of a bit permutation like GIFT, this is essentially always possible for any Sbox when guessing an intermediate round *i* and *j* is 2 or 3 modulo 4, since it will be always XORed with the round key of the following round.

## Generic ideas for improving key guessing: minimal and parallel guesses and efficient merging

In this paper we are going to present how the key-recovery of differential attacks can be improved in a quite generic way, by considering three a priori simple ideas:

- Using the framework from [5], we can reduce the number of needed key bits to guess in each key recovery step, ultimately reducing the number of triplets (and therefore key candidates) generated. We sometimes recover linear relations (*i.e.* with absorption) instead of direct bits, but this relations are also useful for efficient sieving during the posterior merging phase. We will show here how to apply it in the GIFT case.
- Instead of guessing all the needed key bits round per round, we consider subsets of key bits that might be interesting (possibly covering several partial rounds) and guess them in parallel, keeping the partial solutions in lists, in a quite innovative approach.
- Use efficient list merging algorithms, in particular the ones from [6, 12] for recomposing the parallel lists and for finding the well defined triplets to test.

In order to illustrate this, we propose an example on the previously best known attack on GIFT-64, which is a related-key attack reaching 26 rounds, and we show how to reduce the time complexity by a factor of about $2^{10}$ while keeping comparable data and memory.

### Key independent relations: pre-sieving

Suppose we are in the following general scenario. Let $S:\mathbb {F}_2^n \rightarrow \mathbb {F}_2^n$ be an *n*-bit Sbox layer. We want to determine *RK* such that

2 $$\begin{aligned} S(X\oplus RK)\oplus S(\widetilde{X}\oplus RK) \in O \end{aligned}$$

where $O \subset \mathbb {F}_2^n$. If *O* is small enough, we have that $(X,\widetilde{X})$ must satisfy some restriction $\mathcal {R}$ in order for Eq. (2) to be satisfiable, *i.e.*

$$\begin{aligned} (X\oplus RK,\widetilde{X}\oplus RK) \hbox { satisfies Equation }(2) \hbox { for any RK }\implies (X,\widetilde{X}) \in \mathcal {R} \end{aligned}$$

and this restriction is independent of the value of *RK*. This is relevant in the case where the value of $(X,\widetilde{X})$ depends on some previous guess (*i.e.* it is actually a triplet), then one can sieve some key guess candidates (or, equivalently, triplets) based on these restrictions. This remark alone allows to perform the further guess of *RK* on a smaller number of triplets, thus reducing the guessing cost. We call this pre-sieving.

Computing such relations is very simple. They come from the fact that Eq. (2) is satisfied if and only if $(X\oplus RK,\widetilde{X}\oplus RK) \in \nabla _{I \rightarrow O}$. For example, in the case of the GIFT Sbox if $I=\mathbb {F}_2^4$ and $O=\{\texttt {0},\texttt {2}\}$, we know that good pairs cannot have input difference in $I_1=\{ \texttt {1},\texttt {2}, \texttt {4}, \texttt {5}, \texttt {7}, \texttt {8},\texttt {9}, \texttt {c}, \texttt {d} \}$, that is $\nabla _{\delta \rightarrow O}$ is the empty set for $\delta \in I_1$, and all such pairs can be sieved without any need to determine *RK*. In other words, the sieving probability $\pi _1$ of each input differences in $I_1$ is

$$\begin{aligned} \pi _1=\frac{\{x:\pi (x)\oplus \pi ( x\oplus \delta )\in O\}}{16}=0 \end{aligned}$$

where $\delta \in I_1$. Furthermore, when the input difference is $\texttt {6}$, we see that the elements of $\nabla _{\texttt {6}\rightarrow O}$ have most significant bits $(x_2,x_3)={00,10}$, that is $2^{-1}$ pairs having this difference can be sieved regardless of *RK* (which is not mixed with the two most significant bits). Notice that the same happens for input differences $\texttt {a}, \texttt {b}, \texttt {d}, \texttt {f}$, so that $I_2=\{\texttt {6}, \texttt {a}, \texttt {b}, \texttt {d}, \texttt {f}\}$ has sieving probability $\pi _2=2^{-1}$, while no pre-sieving is possible $I_3=\{\texttt {0}, \texttt {3}\}$, that is $\pi _3=1$. In particular, means that the total pre-sieve probability amounts to

$$\begin{aligned} \frac{1}{16} \sum _{\delta \in I_1 } \pi _1+\sum _{\delta \in I_2 } \pi _2+ \sum _{\delta \in I_3 } \pi _3 = \frac{1}{16} \left( 9 \cdot 0 + 5 \cdot 2^{-1} + 2\cdot 1 \right) = 2^{-1.83} \end{aligned}$$

where the factor $\frac{1}{16}$ is given by the assumption that the probability of a pair having a fixed difference $\delta$ is uniform over *I*.

### Merging tables for more efficient sieving

The idea of merging is particularly useful in the case where *X* (and therefore $\widetilde{X}$) can be split into two substates that can be independently determined by two parallel guesses.

More precisely, let $(P,{\tilde{P}})$ be a fixed plaintext pair and *MK* be the *m* master key bits necessary to compute $X=f(MK,P)$. Then splitting *X* means that there exist $X=X_A\oplus X_B$ where $X_A= f_A(MK_A,P)$ and $X_B=f_B(MK_B,P)$ where $MK_A$ and $MK_B$ are respectively $\ell _A$ and $\ell _B$ bits of the master key. Then, let us consider the restrictions these substates need to satisfy, that is let $\mathcal {R}$ such that

$$\begin{aligned} (X\oplus RK,\widetilde{X}\oplus RK) \text { satisfies Eq. } 2 \implies (X_A\oplus X_B, \widetilde{X}_A\oplus \widetilde{X}_B) \in \mathcal {R}. \end{aligned}$$

For simplicity, we are going to write *X*(*MK*) for *f*(*MK*, *P*) and similarly $X_A(MK_A)$ for $f_A(MK_A,P)$ and $X_B(MK_B)$ for $f_B(MK_B,P)$.

Indeed, in most concrete instances, and certainly in the case of SPN ciphers, the states $X_A$ and $X_B$, as well as the sieving relations $\mathcal {R}$ can be split group-wise, e.g. Sbox-wise. Then the algorithms for group-wise efficient merging can be used. Following [6], let the size of $\mathcal {L}_A$ of the possible values of $X_A$ be $2^{\ell _A}$ and that of $\mathcal {L}_B$ be $2^{\ell _B}$. Suppose that the elements of the two lists can be decomposed into *t* groups of size $m_i$ for $\mathcal {L}_A$ and $p_i$ for $\mathcal {L}_B$ and that each group must satisfy the relation $\mathcal {R}_i$ (that is $\mathcal {R}=\mathcal {R}_1\times \ldots \times \mathcal {R}_t$) each of probability $\pi _i$, where $i=1, \ldots , t$. Then we can do a memoryless parallel matching by dividing these groups into three parts of $t_1$, $t_2$, $t-t_1-t_2$ groups each and the two tables can be merged for a time complexity of

$$\begin{aligned} \left( \Pi _{i=1}^{t_1} \pi _i \right) 2^{\ell _B+ \sum _i m_i} + \left( \Pi _{i=t_1+1}^{t_2} \pi _i \right) 2^{\ell _A+\sum _i p_i} + \left( \Pi _{i=1}^{t_1+t_2} \pi _i \right) 2^{\ell _A+\ell _B} \end{aligned}$$

and a memory complexity given by storing the initial and final lists. This is to be compared to the naive cost of guessing all bits to determine *X*, that is $2^{\ell _A+\ell _B}$. In our attack, we will use this to avoid enumerating, for example, a list of $2^{38}$ possible values by merging two tables of $2^{19}$ bits for a cost of $2^{29.68}$.

We remark that *X* can be split into more than two substates and one would simply have to use the algorithm recursively.

## Related-key attack on 26 rounds of GIFT-64 [13]

Since our attack is based on [13], we here give a high level description of how the original attack works. We refer to [13] for details. It uses an 18-round trail with differential probability of $2^{-58}$, that adds 3 rounds in the beginning and 5 in the end.

### The distinguisher

The attack uses a 18-round differential related-key distinguisher. The input/output differences are

$$\begin{aligned} \Delta _{in}=\texttt {0x0060 0000 0006 0000}, \quad \Delta _{out}=\texttt {0x8200 0000 2800 0000}, \end{aligned}$$

while the master key is related by having a difference

$$\begin{aligned} \Delta MK= \texttt {0x0000 0000 0000 0000 0000 0000 0028 0000}. \end{aligned}$$

### The key recovery procedure

The key recovery is done by first organizing the data set into structures and then the key-guess is done using the partial sum technique and taking the properties of the key schedule into account. The goal is to append three rounds of key guessing before the distinguisher and five rounds of key guessing after it.

#### Building the starting pairs

To generate the starting pairs, we prepare structures of $2^{56}$ elements, each containing all possible plaintexts such that only the bits $Y^0[16, 20, 21,$ 25, 33, 40, 44, 45] are fixed, and the corresponding ciphertext of the 26-round encryption. Notice that we can consider $Y^0$ as the plaintext for the sake of simplicity, since the preceding Sbox layer can be uncomputed at no cost. We can then build $2^{112}$ pairs for each two structures that satisfy the difference in $\Delta Y_0$, *i.e.* out of $2^{57}$ encrypted plaintexts. This means that we can prepare *S* twin structures, *i.e.* $N_1= \mathcal {S} \cdot 2^{112}$ pairs can be used in the attack, with a data complexity of $\mathcal {S} \cdot 2^{57}$. In the original attack $\mathcal {S} =2^{3.96}$.

For each possible pair, we obtain the corresponding ciphertext pair and then do the key recovery as follows. We partially guess each round-key Sbox by Sbox and verify whether $\Delta Y^1$ is satisfied, discarding the triplets that do not. As an example, for each possible pair $(X, \widetilde{X})$, we start from the leftmost Sbox and guess the two key bits $k=RK^{0}[0,1]$, so that we can verify for which triplets $(x,\tilde{x}, k)$ the desired condition

3 $$\begin{aligned} S(x\oplus (k_1,k_0,0,0))\oplus S(\tilde{x}\oplus (k_1,k_0,0,0)) \in \{\texttt {0},\texttt {8}\} \end{aligned}$$

is satisfied. Notice that this check costs 2 Sbox evaluations for each of the possible triplets of plaintext pair and key guess, and sieves $2^{-3}$ of them due to Eq. (3). Therefore, this first step will cost $2 \cdot \mathcal {S} \cdot 2^{112} \cdot 2^2 =2\cdot \mathcal {S} \cdot 2^{114}$ in total and will sieve $\mathcal {S} \cdot 2^{114-3}=\mathcal {S} \cdot 2^{111}$ triplets. We then repeat the same procedure for the other nibbles and round keys, each time guessing some bits of the key and then filtering the triplets that do not satisfy the desired output difference.

According to [13], after analysing the first two rounds, if $N_1$ is the number of possible starting pairs, then we will end up with $N_1 \cdot 2^{-56}$ pairs after guessing the earlier rounds, having guessed $2^{40}$ key values, which means we will have $N_1\cdot 2^{-56+40}=N_1 \cdot 2^{-16}$ triplets after guessing the earlier rounds.

After that, we look at the tail of the encryption and guess 24 bits of $RK^{25}$, which also consists in the bottleneck of the guessing of the final rounds, when $N_1 \cdot 2^{-16+24}=N_1 \cdot 2^8$ triplets are generated. Since all the active bits of $RK^{24}$ were already determined when guessing the earlier rounds, we can simply determine $X^{22}$ by 2 full round encryptions (or approximately $2\cdot 32$ Sbox computations) with no further guess. This step alone will cost $N_1 \cdot 2^{8+6}=N_1 \cdot 2^{14}$ Sbox computations. Finally, the remaining bits of $RK^{23}$ and $RK^{22}$ are guessed similarly to $RK^{0}$ and $RK^{1}$, for a total complexity of the entire guessing of $N_1\cdot 2^{15.9}$ Sbox computations, which corresponds to $T_1=N_1\cdot 2^{15.9} \cdot \frac{1}{16\cdot 26} = \mathcal {S}\cdot 2^{123.16}$ 26-round encryptions.

To estimate the data complexity needed for a success probability of the attack of at least 0.9, the authors have used the model provided in [3], so that the choice of $\mathcal {S}=2^{3.96}$ and the choice of a threshold $\theta =2$ result in a false alarm probability $\beta = 2^{-9.14}$, meaning that the total complexity of the attack is

$$\begin{aligned} T_1+2^{128}\cdot \beta (1-2^{-64}) \approx 2^{123.23} \end{aligned}$$

for a data complexity of $2^{60.96}$ and memory complexity is $2^{102.86}$ given by storing the generated subkeys.

In the following section we are going to see how to improve $T_1$ by a factor of $2^{12.24}$, which will lead to different trade-offs for time and memory complexity. We are also going to see how the significant reduction of the number of generated triplets leads to the possibility of a significant improvement in the memory complexity.

## Improved related-key attack on 26-round GIFT

In this section we present how to improve the key-recovery and therefore decreasing the time complexity of the attack. We also discuss a second method to do the key-recovery which, at the cost of a smaller improvement in time complexity, significantly decreases the memory complexity as well.

### A high level overview of the guessing steps

Similarly to the original attack presented in the previous section, we prepare $\mathcal {S} \cdot 2^{57}$ plaintexts divided in *S* structures.

In order to build the good triplets through the first round, for all $\mathcal {S} \cdot 2^{57}$ plaintexts *x*, we generate the partner $\tilde{x}$ such that $S(x)\oplus S(\tilde{x})$ is in the desired set of differences by guessing the necessary amount of key bits. To do this, we guess the necessary key material based on the value of *x*, which is usually significantly less than guessing both bits of key.

The main idea for this step is that a pair $(x\oplus k, x\oplus \delta \oplus k)$ is analogous to the one used to compute the pre-sieving introduced in Sect. 3, that is, since a good pair if and only if $x\oplus k$ belongs to $\nabla _{I \rightarrow O}$ and this set is usually small for *GS*, the restrictions in the two least significant bits (which are not mixed with any key material) result in the possibility of filtering out plaintexts (or triplets) without any key guess or determination. Another important consequence of the small size of $\nabla _{I \rightarrow O}$ is that in most case the sole knowledge of the two least significant bits is enough to completely determine the entire word $x\oplus k$ and, therefore, the value of *k*. Finally, we will also use the techniques reminded in Sect. 2.3 for a finer key guessing and generate a smaller amount of triplets.

In this way, we first guess and determine $RK^0$, generating $2^{N_1-18.62}$ triplets and then do the same for $RK^1$, generating $2^{N_1-21}$ triplets. The bottleneck of this step is the analysis of the $2^{N_1-18.62}$ triplets at the beginning of the guess of $RK^1$, which is about $2^{N_1-17.62}$ Sbox computations. We remark that, as a result of the better sieving, we have generated $2^{-5}$ less triplets than with the simple partial sum technique. This reduced guess comes at the cost of some master key bits not being determined at this point, or being only partially determined via linear relations, which will be crucial to keep track of in order to reduce the guessing material for the last rounds, where the same key bits will need to be determined.

In fact, for each of the generated triplet, we then go to the tail of the encryption and do the guessing of the final round keys $RK^{24}, RK^{25}$ from the ciphertext side. However, our goal here is to reduce the amount of key guessing by determining in parallel two separate halves of the state $X^{24}$ (that can be determined by independent key guesses) in order to exploit the fact that the differential transitions that need to be satisfied in round 23 allow for a sieving independent of the value of $RK^{23}$, thanks again to the fact that $\nabla _{I\rightarrow O}$ is small. The idea is that we can build two tables with the possible values that the two states of $X^{24}$ can take depending the key guesses and then merge them using the fact that the resulting full state must satisfy some conditions given by $\nabla _{I\rightarrow O}$. Notice that for correctly estimating the complexity of this step, it will be crucial to determine which key guesses will be needed for determining each table.

Finally, we will guess $RK^{23}, RK^{22}, RK^{21}$ by guessing group of nibbles in a specific order that allows to keep the number of triplets low at each step, thanks to the possibility of pre-sieving in those rounds. This step will have a complexity of $2^{8.75}$ for each triplet.

Notice that the above key recovery strategy can be concretely applied in two different ways, providing two possibly different trade-offs (discussed in Sect. 5.4):

1. by building all triplets and storing for each triplet the key guess that it determines and then determining the remaining bits for the subkey candidates that are voted by a number of triplets above a certain threshold $\theta$. The memory complexity given by the storage of the possible sub key candidates.
2. by generating each triplet one by one and, for each guess the remaining undetermined bits of key so that the full master key can be tested.

### Guessing $RK^0$ and $RK^1$

We here provide a detailed description of how to do a better sieving than the one used in [13] that was explained in Sect. 4.2, by using the fact that the two least significant bits of each input nibble are always known, and that sometimes to determine good pairs (triplets), the guessing of some key bits is unnecessary or can be postponed thanks to the key-guessing techniques presented in Sect. 2.3. It is especially relevant to keep track of these bits, because, when guessing the last round keys, we are going to make use of the partial knowledge of the master key accumulated in the earlier rounds. For this reason, for each generated triplet, we will have to keep track of which bits were or were not determined in the previous key-guess.

**Fig. 1:**
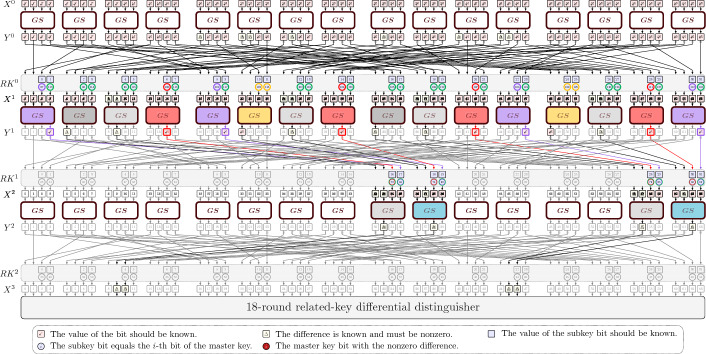
Circled in green the key bits that are determined or guessed for each triplet; in violet the ones that may be undetermined, coming from Type 1; in red the ones that may be undetermined, coming from Type 4; in yellow the ones that may be undetermined, coming from Type 5; in blue the ones that may be undetermined, coming from Type 6. Sboxes are counted from left (Color figure online)

We first provide a detailed example of how to do the key-guessing of the leftmost Sbox, and then we will analyse each Sbox based on the transition that they have to satisfy (see Fig. 1). For example, Type 1 Sboxes are the ones satisfying $XXXX \rightarrow 000X$.

#### Detailed key-guessing for Type 1 Sbox

Let us now see an example of how to generate a smaller number of triplets than what was done in [13], by reducing or postponing the amount of guessed key material. Consider again the leftmost Sbox, for which a differential transition of the kind $XXXX \rightarrow 000X$ has to be verified, *i.e.* the input pairs can take any value and the output pair must satisfy a difference of $\texttt {0}$ or $\texttt {8}$. Furthermore, we will determine the most significant output bit $y_3$ because it will be needed to check the transitions in the next round. In reality, determining a linear relation on one key bit may suffice to avoid an unnecessary guess thanks to the key absorption technique.

In order to generate the good triplets, we first notice that the possible input differences for this transition are $\mathcal {D}=\{\texttt {0},\texttt {5},\texttt {7},\texttt {9},\texttt {b},\texttt {d},\texttt {f}\}$. As explained in Sect. 3, this implies that for each $x\in \mathbb {F}_2^4$, not all $(x,\tilde{x})$ need to be considered, but simply $(x,x\oplus \delta )$ for $\delta \in \mathcal {D}$.^2^ Furthermore, we can sieve on average half of the pairs $(x, x\oplus \delta )$ where $\delta \in \{\texttt {9},\texttt {b},\texttt {d},\texttt {f}\}$ just by looking at the two least significant bit of *x*. In fact, for each such $\delta$, half of the possible values of *x* will not lead to the desired output difference regardless of the value of the key.

As an example, if the input difference is $\texttt {9}$, we know that $\nabla _{\texttt {9}\rightarrow \texttt {8}}=\{\texttt {7},\texttt {e}\}$. Therefore, the possible input values of the Sbox that lead to an output difference $\texttt {8}$ can only be (0111, 1110) or (1110, 0111). In particular, we discard any *x* such that the two least significant bits (which are *not* XORed with any key material) are different from 10 and 11. After that, the only key surviving will be the one that satisfies

$$\begin{aligned} x\oplus (0,0,k_1,k_0)={\left\{ \begin{array}{ll} 0111 &  \text { if } (x_1, x_0)=01 \\ 1110 &  \text { if } (x_1, x_0)=11 \end{array}\right. } \end{aligned}$$

(we remind that $x=(x_3,x_2,x_1,x_0)$ is the 4-bit partial state before the xor of the key, and $k=(k_1,k_0)$ is the partial round-key, so that $(0,0,k_1,k_0)=(0,0,RK^0[0,1])$ in this case). This means that half of all possible pairs $(x,x\oplus \texttt {9})$ will be pre-sieved, and for each of these only one keyword will be possible, generating exactly one good triplet.

The same happens for $\texttt {b},\texttt {d},\texttt {f}$, that is half of the input pairs with the given input difference can be discarded, while the other half fully determines the key bits $RK^0[0,1]$ based on the value of the two least significant bits of the input, each generating one good triplet.

For the pairs with input differences $\texttt {5}$ and $\texttt {7}$, no pre-sieving is possible but both key bits are uniquely determined, again generating exactly one good triplet.

For the pairs of input difference $\texttt {0}$, all of them are necessarily good pairs and we simply need to determine the output value $y_3$. To do so, we always guess one key bit ($k_0$), and then in half of the cases the output is determined and we have one undetermined key bit ($k_1$), in the other we will avoid the guess with key absorption. In particular, each pair will generate 2 triplets with undetermined $k_1$, but for half of them we know that, if in the second round we want to determine $z_0\oplus \kappa _0=y_3\oplus k_1 \oplus \kappa _0$, we can guess simultaneously the value $k_1 \oplus \kappa _0$ (we recall here, from notation, that $\kappa _0$ represents the bit added to the output in the next round). More concretely, we will not guess $k_1$ (which correspond to master key bit 97) as we do not need it, and when guessing the Sbox 3 of the second round, we will directly guess $k_1\oplus \kappa _0$, which corresponds to determining a relation for *MK*[89, 97], that is between master key bits *MK*[97] and *MK*[89].

The above information is summarised in Table 2 where, for each possible input difference, the set of possible good pairs $\nabla _{\delta \rightarrow \texttt {8}}$ are listed, together with the amount of pre-sieve and key sieve that this set determines, followed by the number of triplets it generates. In order to compute the total sieve, or amount of key guesses, or triplets, one has to sum all the values in the respective column weighted by $\frac{1}{16}$ (that is the probability that a random pair, or triplet, has the given input difference).

Overall, at the end of the analysis of this Sbox we will have generated for each possible *x*, $4\cdot 2^{-1}=2$ triplets with differences $\texttt {9}, \texttt {b},\texttt {d},\texttt {f}$ and $2\cdot 1=2$ from the second group of non-zero differences $\texttt {5},\texttt {7}$, all with both key bits determined. We will also have generated 2 triplets of difference $\texttt {0}$, one with $k_1$ undetermined, and another one with $k_1$ will be absorbed. In other words, we generate $6 \approx 2^{2.59}$ triplets for each possible *x*, of which

- $\frac{2}{3}$ have both key bits determined (corresponding to master-key bits 97, 113),
- $\frac{1}{3}$ have $k_1$ undetermined (master-key bit *MK*[97]) of which $\frac{1}{6}$ with possibility of absorbing it when guessing the next round key. This means that, to determine the LSB of Sbox 10 in round 1, we determine neither $k_1$ (*MK*[97]) nor $\kappa _0$ (*MK*[89]), but a linear relation between the two $k_1 \oplus \kappa _0$. This distinction will be later useful when guessing the last rounds.

Notice that the naive key guessing would require to generate for each $x\in \mathbb {F}_2^4$, $2^4$ pairs and $2^2$ key guesses, that is $2^4 \cdot 2^2=2^{6}$ triplets that need $2\cdot 2^6=2^7$ Sbox evaluations to generate $2^{6-3}=2^3$ good ones. In total, $\mathcal {S} \cdot 2^{57} \cdot 2^7=\mathcal {S} \cdot 2^{64}$ Sbox evaluations. However, with our method, each plaintext (or pair) generates on average $6=2^{2.59}$ triplets requiring a complexity of less than 2 Sbox computations per input triplet. Indeed, all the information needed for determining whether a pair is good or not for a certain transition $I \rightarrow O$ can be stored in a table that has at most 16 entries for each possible input difference, each of which has a maximum of 4 possible entries (*i.e.* the elements of $\nabla _{\delta \rightarrow O}$, which correspond to the possible assignments of $x \oplus k$); a lookup to this table costs then less than two consecutive 4-bit Sbox evaluation. Notice that this is in contrast to the naive method, where the number of Sbox evaluations depends on the number of generated triplets (as any possible combination of plaintext pair and key must be tested), whereas our method can directly generate a list of triplets satisfying the desired condition for each input triplet. The total complexity for this step is then $\mathcal {S} \cdot 2^{58}$ Sbox evaluations.

**Table 2:** Triplet sieving for Type 1 ($XXXX \rightarrow 000X$). The triplet sieve takes into account the key absorption used for $\delta =0$

Input difference	$\nabla _{\delta \rightarrow \Delta }$	Pre sieve	# of possible keys	# of triplets
0	$\mathbb {F}_2^4$	$2^{0}$	$2^1$	$2^{1}$
5	$\{ \texttt {9}, \texttt {a}, \texttt {c}, \texttt {f} \}$	$2^{ 0}$	$2^{ 0}$	$2^{ 0}$
7	$\{ \texttt {1}, \texttt {3}, \texttt {4}, \texttt {6} \}$	$2^{ 0}$	$2^{ 0}$	$2^{ 0}$
9	$\{ \texttt {7}, \texttt {e} \}$	$2^{-1}$	$2^{ 0}$	$2^{-1}$
b	$\{ \texttt {0}, \texttt {b} \}$	$2^{-1}$	$2^{ 0}$	$2^{-1}$
d	$\{ \texttt {5}, \texttt {8} \}$	$2^{-1}$	$2^{ 0}$	$2^{-1}$
f	$\{ \texttt {2}, \texttt {d} \}$	$2^{-1}$	$2^{ 0}$	$2^{-1}$

#### Guess of $RK^0$

We will analyse each transition type, and keep track of which bits we do not need to determine, since we might need to guess those bits in the final rounds. Furthermore, with the key-absorption technique we will determine relations on the key bits that will also turn out to be useful in the last rounds. This information is summarised in Fig. 1, where the key bits that will be determined (or guessed) for any triplet are in green, while the ones that are not determined with some probability are in red, and in yellow the ones that are not determined but a linear relationship among them exist.

Type 1 (Sboxes 1, 5, 12, 16) As already discussed, for these Sboxes we can determine both key bits whenever the pair has a non-zero difference. When the pair has difference zero (on average, $\frac{1}{3}$ of the total number of good triplets), then we always guess $k_0$. This is enough in order to determine $y_0$ for half of the pairs, while for the remaining half we can do key-absorption and compute $y_0$ as linearly dependent on the undetermined bit $k_1$.

This means that, on average, for $\frac{1}{3}$ of the total number of good triplets, $k_1$ is undetermined (which correspond to master key bits *MK*[97, 101, 108, 112]) and in half of those cases we do key-absorption between $k_1$ and $\kappa _0$, that is a linear relationship between the master key bits *MK*[90, 101], *MK*[89, 97], *MK*[108, 95], *MK*[112, 96] will be determined when guessing round 1. The possibly undetermined key bits from Type 1 are violet.

The analysis of each of these Sboxes sieves on average $2^{-1.41}$ of all possible triplets for each Sbox, costing 2 Sbox evaluation per triplet. This is to be compared to the naive sieve of $2^{-1}$ costing $2\cdot 2^2 = 2^3$ Sbox computations per triplet.

Type 2 (Sboxes 2, 9) As summarised in Table 4, we determine both key bits for all. In this way, we have a sieving of $2^{-2}$ triplets for each Sbox, which is equivalent to the naive sieve, but costing 2 Sbox evaluations against $2^3$.

Type 3 (Sboxes 3, 7, 10, 14) For every input difference, no pre-sieving is possible but both key bits are then completely determined (Table 5). No additional triplets are then generated, *i.e.* the sieving amounts to a factor of 1.

Type 4 (Sboxes 4, 8, 11, 15) For this group of Sboxes (Table 6, we know that the LSB of the key $k_0$ is always determined, while $k_1$ is undetermined when $\delta =\texttt {6}$ (which happens for $\frac{1}{11}$ of the good triplets), or when $\delta =\texttt {0}$ ($\frac{4}{11}$ of the good triplets), in which case half of the times $k_1$ is also undetermined (it is not needed to determine $y_1$), and in the other half of the cases a key-absorption between $k_1$ and $\kappa _1$ is possible.

Overall, in $\frac{5}{11}$ cases (*i.e.* triplets whose input difference is $\texttt {0}$ or $\texttt {6}$) $k_1$ is undetermined (which correspond to master key bits *MK*[100, 104, 107, 111]), but in $\frac{2}{11}$ cases key absorption is done and we also have bits *MK*[73, 74, 79, 80] undetermined. In this case, however, as soon as the guessing of round 1 will be done, a linear relation between $k_1$ and $\kappa _1$ will be determined, as already noted. In terms of master key bits, a relation between bits *MK*[100, 73], *MK*[104, 74], *MK*[107, 79], *MK*[111, 80] will be determined when guessing the next round.

The possibly undetermined key bits from Type 4 are red. The amount of sieving is $2^{-1.54}$ triplets for each Sbox.

Type 5 (Sboxes 6,13) In this case (Table 7), we know that when $\delta \ne \texttt {0}$, then we fully determine $k_0$ and $k_1$. When the pair has input difference $\texttt {0}$ (which are $\frac{1}{3}$ of the good triplets), following the tree 3 for $y_3$ (Appendix A), then either $k_0$ or $k_1$ are not guessed (with probability $\frac{1}{4}$ and $\frac{1}{2}$ resp.), while in the remaining $\frac{1}{4}$ of the cases $k_0 \oplus k_1$ must be guessed, which means that in this case $k_0$ and $k_1$ are undetermined but a linear relation between the two is guessed.

Overall, this means that $\frac{1}{3}$ of the good triplets for each Sbox, one bit between *MK*[109, 125] and/or one bit between *MK*[102, 118] is undetermined.

The possibly undetermined key bits from Type 5 are depicted in yellow. The total sieving of triplets is $2^{-1.41}$ for each Sbox.

Overall sieve The above method used to guess $RK^0$ generates $2^{- 4 \cdot 1.41 - 2 \cdot 2 - 4 \cdot 1.54 - 2 \cdot 1.41}=2^{-18.62}$ triplets for each pair, for a total of $\mathcal {S} \cdot 2^{93.38}$ triplets at the end of the first round. For comparison, in the original paper [13], $\mathcal {S} \cdot 2^{98}$ triplets are generated.

#### Guess of $RK^1$

Type 6 (Sboxes 10, 16) For this group of Sboxes, when $\delta =\texttt {6}$ (*i.e.* $\frac{1}{7}$ of the cases) the input bit $z_2$ after round key addition is undetermined. This means that bit *MK*[74] (or a linear relations between key bits *MK*[111] and *MK*[80], for the triplets for which key absorption was done in the previous round) is unknown in $\frac{1}{7}$ of the cases. Similarly, bit *MK*[80] (or a linear relation between key bits *MK*[104] and *MK*[74]) is not determined in $\frac{1}{7}$ of the cases. However, $z_3$ is always determined and this means that master key bits *MK*[96] and *MK*[90] (or, when key absorption was done previously, linear relations between key bits *MK*[96, 112] and between key bits *MK*[90, 101], respectively) are also determined. The sieving is $2^{-1.19}$ for each Sbox.

Type 3 (Sboxes 9, 15) For these Sboxes, all the key bits are determined for any triplet, and the sieving is 1. Therefore, key bits *MK*[73, 89] (or linear relations between key bits *MK*[97, 89] and between key bits *MK*[73, 100], respectively), as well as key bits *MK*[79, 95] (or linear relations between masterkey bits *MK*[79, 107] and *MK*[95, 108]) are always determined.

### Guessing the final rounds

From the above considerations, we see that the number of triplets after guessing the early rounds is equal to $N_1 \cdot 2^{-18.62 - 2.38}= N_1 \cdot 2^{-21}$ (instead of $N_1 \cdot 2^{-16}$ of the original paper).

**Fig. 2:**
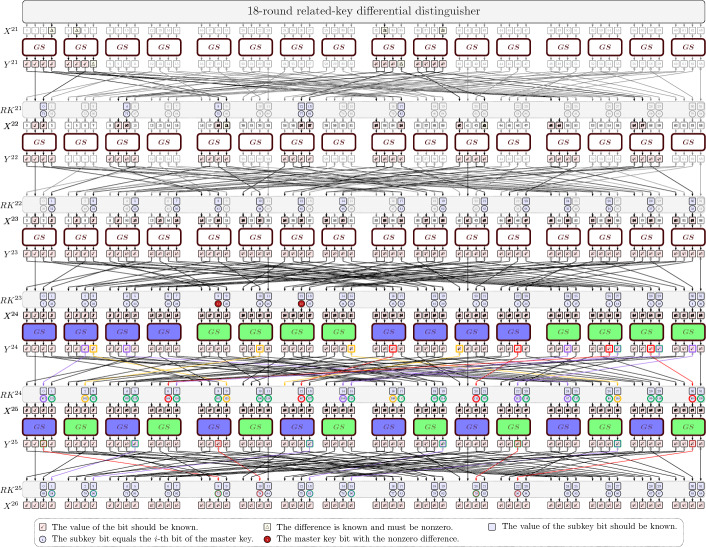
Circled in green the key bits that were always determined/guessed in the earlier rounds; in violet the ones that may be undetermined (coming from Type 1); in red the ones that may be undetermined (from Type 4); in yellow the ones that may be undetermined (from Type 5); in grey the ones that may be undetermined (from Type 6). Sboxes are counted from left (Color figure online)

#### Guess of $RK^{25}$ and $RK^{24}$

The goal is to guess the remaining bits of $RK^{24}$ and $RK^{25}$ (the ones that have not been determined so far) in two separate groups in parallel. We will then merge these two groups of guesses thanks to relations that the state $X^{24}$ needs to satisfy, in order for a good triplet to satisfy the difference $\Delta X^{23}$. In fact, we separately guess the key bits involved in the independent computations in the group of nibbles 1, 2, 3, 4, 9, 10, 11, 12 of the state $X^{24}$ (blue Sboxes) that we call A and nibbles 5, 6, 7, 8, 13, 14, 15, 16 of the state $X^{24}$ (green Sboxes) that we call B (Fig. 2).

Thus, we generate two lists of triplets, one associated to the possible values of the group A nibbles of $X^{24}$ and the other to the possible values of the group B, that we can merge together to determine the full state of the triplets at $X_{24}$. This merging is done considering the filtering of probability $2^{-0.91}$ (or $2^{-0.87}$) per Sbox that are possible in round 23 without knowledge of $RK^{23}$, as well as the linear relations (determined when guessing the first two rounds) between master key bits that are each needed when building a different table (we will explain this in detail).

In fact, similarly to the pre-sieving that was done to filter triplets regardless of the key values, Tables 9 and 10 show that it is possible to filter out triplets for the transitions $XXXX\rightarrow X0X0$ and $XXXX \rightarrow 0X0X$ respectively, simply by looking at the possible values that the first two bits of $X^{24}$ can have in order to belong to the desired $\nabla _{\delta \rightarrow \Delta }$ (where $\Delta$ is *X*0*X*0 or 0*X*0*X*).

In order to determine how many key bits must be guessed for building each table, we keep track of which key bits were already determined in the earlier rounds.

The following 20 key bits of the masterkey that are part of $RK^{24}$ are always guessed or determined in the first two rounds: $MK[121, 122, 103, 123, 124, 105, 106, 126, 127, 128, 113, 110, 114,$ 115, 116, 117, 98, 99, 119, 120]. We now focus on the key bits that may be undetermined for building each table, summarized in Table 3.

**Table 3:** Key bits determined in the earlier rounds (bits 73 and 74 are determined up to a linear relationship)

Group	Master key bits involved	Notes
A	*MK*[81, 82, 75, 83, 76, 84, 65, 66, 67, 91, 68, 92]	All bits undetermined with probability $p=1$
	*MK*[89, 97], *MK*[90, 101], *MK*[109, 125]	One out of each couple of bits undetermined with $p=\frac{1}{3}$
	$MK[111, 117, 73^*, 74^*]$	Each bit undetermined with $p=\frac{5}{11}$
B	*MK*[69, 93, 77, 85, 70, 94, 78, 86, 71, 87, 72, 88]	All bits undetermined with probability $p=1$
	*MK*[96, 112], *MK*[95, 108], *MK*[102, 118]	One out of each couple of bits undetermined with $p=\frac{1}{3}$
	*MK*[100, 104, 79, 80]	Each bit undetermined with $p=\frac{5}{11}$

Building the first table for Group A In order to determine $X^{24}$ in the first group of nibbles, the following 12 bits from $RK^{25}$ are always undetermined and need at this point to be guessed: *MK*[81, 82, 75, 83, 76, 84, 65, 66, 67, 91, 68, 92].

As for the remaining bits involved in the determination of the bits of Group A, we have already seen in the previous section that one bit out of master key bits 89 and 97 is undetermined with probability $\frac{1}{3}$, while being otherwise fully determined; similarly, one bit out of master key bits *MK*[90, 101] is undetermined with probability $\frac{1}{3}$ and one bit out of the master key bits *MK*[109, 125] is also undetermined with probability $\frac{1}{3}$. Furthermore, bits *MK*[111, 107] are each undetermined with a probability of $\frac{5}{11}$, while bits *MK*[73, 74] are each undetermined when key-absorption happened for Sboxes 4 and 8 of round 0, *i.e.* with a probability of $\frac{2}{11}$. However, for simplicity we suppose it happens with probability $\frac{5}{11}$, so that we can consider one single case.

The above information, and the equivalent for Group B, is summarised in Table 3.

Building the second table for Group B The following 12 bits from $RK^{25}$ are always undetermined and need to be always guessed: *MK*[69, 93, 77, 85, 70, 94, 78, 86, 71, 87,  72, 88]. However, only one bit out of master key bits *MK*[96, 112], as well as one bit out of *MK*[95, 108], each need to be guessed with probability $\frac{1}{3}$; similarly, one bit out of *MK*[102, 118] is undetermined with probability $\frac{1}{3}$. On the other hand we consider bits 100, 104, *MK*[79, 80] undetermined each with probability $\frac{5}{11}$ for simplicity, as before.

**Linear relations from key-absorption** Finally, whenever key-absorption was done in round 0 for Sboxes 13, 9, 6, 2 (Type 4), linear relations between key bits were possibly determined, but are not possible to be used when building tables because the relations are between key bits that are needed for different tables (contrary to the linear relations among bits belonging to the same group, which we have indeed used). These linear relations can however be used when merging the tables. In particular, a relation between master key bits *MK*[74, 104] and *MK*[80, 111] are known each with probability $\frac{6}{7}$ (*i.e.* when $x_1\oplus \kappa _1\oplus k_0$ was determined in round 1) when key absorption was done on Sboxes 8 and 15 of round 0. Similarly, a linear relation between bits *MK*[73, 100] or between bits *MK*[79, 107] is known always whenever key absorption was done on Sboxes 13 and 6 of round 0.

**How to recover** $X^{24}$ **merging the tables efficiently** After building the tables $\mathcal {L}_A$ and $\mathcal {L}_B$ from Group A and Group B respectively, with entries equal to a possible substate value of $X^{24}$ according to a different key guess, we want to finally merge the tables to recover $X^{24}$.

In order to merge them efficiently, we consider $t_1$ Sboxes from the first group and $t_2$ sboxes from the second group (e.g., when $t_1=t_2=4$ we can consider Sboxes 1, 2, 3, 4 and Sboxes 5, 6, 7, 8) and do the merging of the tables according to these Sboxes, following the memoryless parallel matching introduced in [6].

In particular, let us consider the 6 bits of each Sbox that count towards the sieve, that is the key-independent input bits of each input pair ($x_3, \tilde{x}_3, x_2, \tilde{x}_2$) and the two output bits of the difference ($z_1=\Delta y_3, z_0=\Delta y_1$). We then do a memoryless group-wise parallel matching, considering for each Sbox the group of three bits $x_3, \tilde{x}_3, z_1$ and the *m* bits of the linear relation of the key ($3\cdot t_1+m$ bits in total) and the group of three bits of $x_2,\tilde{x}_2, z_0$ ($3\cdot t_2$ bits in total), that is we decompose the two lists according to the values of groups of $3\cdot t_1+m$ and $3\cdot t_2$ bits respectively.

More precisely, let $\mathcal {L}_A$ and $\mathcal {L}_B$ be of size $2^{\ell _A}$ and $2^{\ell _B}$ respectively, ordered lexicographically according to the first group of $3\cdot t_1$ and the possible $2^m$ values of the key bits involved in the linear relations previously determined, and then to the second group of $3\cdot t_2$ bits. That is, the first $2^{\ell _A-3\cdot t_1-m}$ entries of $\mathcal {L}_A$ are all 0 in the aforementioned $3\cdot t_1+m$ bits, 1 in the next $2^{\ell _A-3\cdot t_1-m}$, and so on, so that $\mathcal {L}_A$ is composed of sublists $\mathcal {L}(\alpha )$ whose first $3\cdot t_1$ bits take value $\alpha$ for all $\alpha \in \{0, \ldots , 2^{3 \cdot t_1+m}-1\}$. These sublists are of size $2^{\ell _A-3\cdot t_1}$. The same we do for $\mathcal {L}_B$, being an ordered list of sublists $\mathcal {L}_B(\beta )$ for $\beta \in \{0, \ldots , 2^{3 \cdot t_1+m}-1\}$.

Then for all possible $\alpha \in \{0, \ldots , 2^{3 \cdot t_1+m}-1\}$, we do the following

1. we consider the sublist $\mathcal {L}_A(\alpha )$ and build a new list $\mathcal {L}_{aux}$ made by the union of $(\alpha , \mathcal {L}(\beta ))$ such that the relation given by the sieve is satisfied. This happens on average for $2^{t_1\cdot (3-0.91)}$ such $\beta$, so that $\mathcal {L}_{aux}$ has size $2^{t_1\cdot (3-0.91)+\ell _B-3\cdot t_1}= 2^{\ell _B-0.91\cdot t_1}$ and is then sorted according to the second group of $3\cdot t_2$ bits. This step has approximately the cost of building $\mathcal {L}_{aux}$, that is $2^{\ell _B-m-0.91\cdot t_1}$;
2. for each element in $\mathcal {L}_A(\alpha )$, we check whether it belongs to $\mathcal {L}_{aux}$ and can be associated. This step has the cost of finding these matches, which is approximately $2^{\ell _A-3\cdot t_1-m+(3-0.87)\cdot t_2}$, since $\mathcal {L}_{aux}$ was ordered according to the group of $3\cdot t_2$ bits.

After that, for each finding, we can test and sieve with respect to the remaining group of bits and to the linear relations for a cost of $2^{\ell _A+\ell _B-m-t_1\cdot 0.91-t_2\cdot 0.87}$ and a further sieve of $2^{(8-t_1)\cdot 0.91-(8-t_2)\cdot 0.87}$. The total complexity is then on average approximately

4 $$\begin{aligned} \begin{aligned}&2^{m+3\cdot t_1}\cdot (2^{\ell _B-m-0.91\cdot t_1}+2^{\ell _A-m-3\cdot t_1+(3-0.87)\cdot t_2})+2^{\ell _A+\ell _B-m-t_1\cdot 0.91-t_2\cdot 0.87}\\&\quad = 2^{\ell _B-2.09\cdot t_1} + 2^{\ell _A-2.13\cdot t_2}+2^{\ell _A+\ell _B-m-0.91\cdot t_1- 0.87\cdot t_2} \end{aligned} \end{aligned}$$

Let us now see how to estimate the above complexity for the average key guess, *i.e.* depending on the sizes of the lists and the number of linear relations due to the previous key guessing.

Computing the expected complexity of the merging Let us see as an example the worst scenario, and then we will examine the general case. The worst possible situation happens when all bits that may have not been determined or guessed in the earlier rounds, are indeed undetermined and need to be guessed at the moment of building the tables for $X^{24}$. This correspond to the case when all the Type 1 transitions have one key bit undetermined (probability $(\frac{1}{3})^4$, as well as Type 5 ($(\frac{1}{3})^2$) and Type 6 ($(\frac{1}{7})^2$); finally, for all Type 4 key-absorption was performed ($(\frac{5}{11})^4$). This then happens with a probability:

$$\begin{aligned} \left( \frac{1}{3}\right) ^6\cdot \left( \frac{1}{7}\right) ^2\cdot \left( \frac{5}{11}\right) ^4=2^{-19.67}. \end{aligned}$$

In this case, we would have 19 bits to guess in order to build each table, *i.e.* for each pair we would have to merge two tables of size $2^{19}$ for which 16 (non-linear) relations exist, each satisfied with a probability of $2^{-0.91}$, and two linear relations coming from the key-absorption of Sboxes 13 and 6 of round 0 (Sboxes 9 and 2 of round 0 do not yield a relation because we are in the worst case scenario in which the Type 6 Sboxes of round 1 do not determine $x_1$). Therefore, the worst case scenario (which corresponds to the case where $\ell _A=\ell _B=19$, $m=2$ and happens with probability $2^{-19.67}$) has a time complexity according to Eq. (4) equal to

$$\begin{aligned} 2^{19+2.09t_1}+2^{19+2.13t_2}+2^{36-0.91\cdot t_1- 0.87\cdot t_2} \end{aligned}$$

which, for the case of $t_1=t_2=4$ is equal to $2^{29.68}$.

Let us show how to determine the average complexity of the above procedure. As summarised in Table 3, we always need to guess 12 bits for each table, while the remaining 7 may be determined, depending on the guessing of the earlier rounds. Let $L_A=\ell _A-12$ be the random variable that counts the number of guesses necessary to build the first table, in addition to the 12 which are always necessary; similarly, let $L_B=\ell _B-12$ be the number of guesses for the second table and let *M* be the number of linear relations from Type 4 transitions coming from key-absorption and that can thus only be used *during* the merging. To keep track of this, we model each Sbox as a random binomial variable.

For Sboxes of Type 1 and 5 the variable takes 0 when both key bits of the Sbox are determined, while 1 when they are not (we have seen that this can considered always as one bit known, one bit undetermined). We call these variables $X_1, X_5, X_6, X_{12}, X_{13}, X_{16}$ (the index comes from the number of the respective Sbox in round 0) and they are then binomial variables which are 1 with probability $\frac{1}{3}$. Similarly with Sboxes of Type 4, they can take 0 with probability $\frac{6}{11}$, but the variable takes 1 when we are doing key-absorption and then two bits are undetermined (so with probability $\frac{5}{11}$, in the simplified worse scenario). The variables are named as $Y_4, Y_8, Y_{11}, Y_{15}$. A linear relation is however known in the case of Sboxes 13 and 6 of round 0, while for Sboxes 9 and 2 it is only known when the random variably of Sboxes 7 and 1 of round 1 (which we call $Z_{10}$ and $Z_{16}$) take value 0.

Therefore, we have 12 random binomial variables, mutually independent, and depending on the value of each we need to guess $$12+L_A$$ key bits for Table A and $$12+L_B$$ bits for Table B, and have at our disposal *M* additional linear relations coming from key-absorption. For example, in the worst case scenario, we have seen that $$L_A=L_B=7$$ and $$M=2$$. Or when $$ Z_{10}$$ and $$Z_{16}$$ are 0, but $$X_i$$ and $$Y_j$$ are 1 for all $$i=1,5,6,12,13,16$$ and $$j=4,8,11,15$$ (which happens with probability $$2^{-14.50}$$): then $$L_A=L_B=7$$ and $$M=4$$. Another possibility is, for example, if $$Z_{10}$$ $$Z_{16}$$ and $$Y_{15}$$ are 0, and $$X_i$$ and $$Y_j$$ are 1 for all $$i=1,5,6,12,13,16$$ and $$j=4,8,11$$ (which happens with probability $$2^{-14.24})$$: then $$L_A=6, L_B=7, M=3$$.

From Eq. (4), we know that the complexity of the merging if $L_A=l_A$ and $L_B=l_B$ is

$$\begin{aligned} \mathcal {C}(l_A, l_B, r)=2^{12}(2^{l_A+2.09t_1}+2^{l_B+2.13t_1})+2^{24+l_A+l_B-m-0.91\cdot t_1-0.87\cdot t_2}. \end{aligned}$$

Then, the average complexity is given by

$$\begin{aligned} \sum _{0 \le l_A, l_B \le 7, 0 \le r \le 2} \Pr (L_A=l_A, L_B=l_B, M =m)\cdot \mathcal {C}(l_A, l_B, m). \end{aligned}$$

Using a computer program to compute the above costs and probabilities, we find out that the average complexity of the merging is $2^{24.22}$, where we have used the heuristic choice of $t_1=t_2=3$ when $l_A+l_B\le 10$, while $t_1=t_2=4$ when $l_A+l_B>10$.

At the end of this, an average of

$$\begin{aligned} \sum _{0 \le l_A, l_B \le 7, 0 \le m \le 4} (l_A+l_B-m) \cdot \Pr (L_A=l_A, L_B =l_B, M=m) \end{aligned}$$

key bits are guessed, which we have found to be equal to 28.03 key bits (including the linear relations). Given that a total sieving of probability $2^{-8\cdot 0.91 - 8\cdot 0.87}$ is done, then $T \cdot 2^{28.03-8\cdot 0.91-8\cdot 0.87}=T \cdot 2^{13.79}$ remain on average, if *T* was the number of triplets at the beginning of this step.

#### Guessing $RK^{23}$, $RK^{22}$, $RK^{21}$

At this point of the key guessing, the active bits of $RK^{21}$ are already determined as the master key bits *MK*[71, 73, 75, 89, 77, 91, 93, 95] were already determined when guessing $RK^{25}$. We need then to guess $RK^{23}$ and $RK^{22}$. Let $T^{(0)}$ be the number of triplets at the start of this phase of the guessing.

We divide the guess of $RK^{23}$ into four steps, by determining four different substates of $X^{23}$. First, we guess Sboxes 1, 5, 9, 13 of round 23, each generating respectively $2^{0.91}$, $2^{0.87}$, $2^{0.91}$, $2^{0.87}$ triplets (according to Tables 9 and 10), which in total is $2^{3.56}$ new triplets. Building these triplets can be estimated to cost $2\cdot 4$ Sbox computations per triplet, for a total complexity of $C^{(0)}=8\cdot T^{(0)}$ Sbox computations. Since these values determine nibbles 1 and 3 of $X^{23}$, which both need to satisfy the transition $XXXX\rightarrow 0XX0$, a pre-sieve of $2^{-1}$ is possible for each nibble (Table 13) in round 22, without need of guessing $RK^{22}$. Therefore, at the end of this step $T^{(1)}= T^{(0)}\cdot 2^{3.56-1-1}=T^{(0)}\cdot 2^{1.56}$ remain.

Next, we guess $RK^{23}$ for the Sboxes 3, 7, 11, 15 of round 23, generating again a total of $2^{4.56}$ triplets for a cost of $2\cdot 4$ Sbox computations for each triplet, that is $C^{1}= 8 \cdot T^{(1)} \cdot 8= T^{(0)}\cdot 2^{3.56}$ Sbox computations. This determines nibbles 9 and 11 of $X^{23}$, which need to satisfy the transition $XXXX\rightarrow X00X$ and $XXXX\rightarrow X001$ respectively, so that a pre-sieve is possible of $2^{-0.71}$ and $2^{-1.09}$ respectively (Tables 15 and 16). At the end of this step, $T^{(2)}= T^{(1)} \cdot 2^{3.56}\cdot 2^{-1.90}= T^{(0)}\cdot 2^{1.56+3.56-0.71-1.09}=T^{(0)}\cdot 2^{3.32}$ remain.

Next, we guess Sboxes 2, 6, 10, 14 of round 23, generating once more a total of $2^{3.56}$ triplets for a total cost of $C^{2}= 8 \cdot T^{(2)} =T^{(0)}\cdot 2^{6.32}$ Sbox computations. This determines nibbles 5 and 7 of $X^{23}$, which need to satisfy the transition $XXXX\rightarrow 00X1$ and $XXX\rightarrow 00XX$ respectively, so that a pre-sieve is possible of $2^{-1.19}$ and $2^{-0.68}$ respectively (Tables 11 and 12), after which $T^{(3)}= T^{(2)} \cdot 2^{3.56}\cdot 2^{-1.87}= T^{(0)}\cdot 2^{3.22+3.56-1.87}=T^{(0)}\cdot 2^{5.01}$ remain.

Finally, we guess nibbles 4, 8, 12, 16, generating again a total of $2^{3.56}$ triplets. This costs a total of $C^{3}= 8 \cdot T^{(3)}= T^{(0)}\cdot 2^{8.01}$ Sbox computations and determines nibbles 13 and 15 of $X^{23}$. These two Sboxes need to both satisfy the transition $XXXX\rightarrow XX00$, so that a pre-sieve of $2^{-0.83}$ is possible for each (Table 14). At the end of this step, $T^{(4)}=T^{(3)} \cdot 2^{3.56-1.66}=T^{(0)}\cdot 2^{6.91}$ triplets will remain.

In order to determine $RK^{22}$, we do similarly as before, determining first Sboxes 1, 5, 9, 13, which correspond to generating each $2^{1}$, $2^{0.19}$, $2^{0.71}$, $2^{0.83}$ triplets respectively, for a total of $T^{(4)}\cdot 2^{2.73}$ new triplets and a cost of $C^{4}= 8 \cdot T^{(4)}=2^{9.91}$ Sbox computations. After this, Sbox 16 of round 21 will be fully determined, since the master key bits of $RK^{21}$ were already guessed, and a sieve of $2^{-4}$ is possible, so that $T^{(5)}=T^{(4)}\cdot 2^{2.73-4}= T^{(0)}\cdot 2^{5.64}$ triplets remain.

Finally, we determine the remaining nibbles of $RK^{22}$, which generate for each triplet $2^{1+0.68+0.09+0.83}=2^{2.6}$ new ones, for a total of $T^{(6)}=T^{(5)}\cdot 2^{2.6}= 2^{8.24}$ and a cost of $C^{5}= 2\cdot T^{(5)}=2^{6.64}$ Sbox computations.

After this, the final nibbles 7, 8 and 15 of round 21 are determined, each providing an additional sieve of $2^{-4}$ for each of them since $RK^{21}$ is determined, that is $T^{(6)}\cdot 2^{-12}=T^{(0)}\cdot 2^{-3.76}$ will be the number of triplets generated at the end of the key guessing.

The cost of generating these triplets is then

$$\begin{aligned} \sum _{i=0}^{5} C^{(i)}= T^{(0)}(2^3+2^{4.56}+2^{6.32}+2^{8.01}+ 2^{9.91}+ 2^{5.64})=2^{10.43} \cdot T^{(0)} \end{aligned}$$

Sbox computations.

### Final complexity

Let us estimate the final complexity of the attack with the improved key guessing. We propose two different variants for better time complexity or better memory complexity.

#### Improved time complexity

We first prepare the data in $\mathcal {S}$ structures of size $2^{57}$ plaintext-ciphertext pair each. We will estimate $\mathcal {S}$ later, but for now we observe that this step has a time complexity of $\mathcal {S}\cdot 2^{57}$ 26-round encryptions and the same amount of plaintext ciphertext pairs.

We can then estimate that the complexity of guessing the first round keys $RK^{0}$ and $RK^{1}$ to be approximately the cost of generating the triplets of the guess of $RK^{0}$, that is

$$\begin{aligned} 2 \cdot N_1\cdot 2^{-18.62} =2 \cdot \mathcal {S} \cdot 2^{112} \cdot 2^{-18.62}=\mathcal {S}\cdot 2^{94.38} \end{aligned}$$

Sbox computations. In fact, when guessing $RK^{1}$ we actually reduce the possible triplets thanks to the pre-sieving. All in all, the cost of guessing the first two round key is actually negligible in comparison to the steps necessary to guess the final round keys, as we are now going to explain.

In fact, the next step uses $\mathcal {S} \cdot 2^{112-21}$ triplets ($2^{-21}$ were sieved when guessing the first two rounds as was observed), and for each of them we guess $RK^{24}$ and $RK^{25}$ in two separate groups that are needed to build two tables for $X^{24}$, each of size up to $2^{19}$, that we have seen can be merged for an average complexity of $2^{24.32}$. Therefore, this step alone can be estimated to cost about

$$\begin{aligned} N_1 \cdot 2^{-21}(2^{19}+2^{19}+2^{24.32}) \end{aligned}$$

Sbox computations.

Finally, for each of the newly generated $\mathcal {S} \cdot 2^{112-21} \cdot 2^{13.79}$ triplets we have seen that the guess of $RK^{23}, RK^{22}, RK^{21}$ costs $2^{8.75}$ on average. Therefore, estimating one Sbox computation as $\frac{1}{26\cdot 16}$ 26-round encryptions), the total time complexity of the entire procedure is approximately

5 $$\begin{aligned} \begin{aligned} T_1&=N_1 \cdot 2^{-21}(2^{19}+2^{19}+2^{24.32}+ 2^{13.79}\cdot 2^{10.43}) \cdot \frac{1}{26\cdot 16}= N_1 \cdot 2^{-4.39} \\&= \mathcal {S} \cdot 2^{107.61} \end{aligned} \end{aligned}$$

26-round encryptions. We remark that the total subkey recovery phase with the partial sum technique of the original paper had a complexity of $\mathcal {S}\cdot 2^{119.22}$.

After this phase of the key guessing, we will count how many triplets vote a subkey value $\tilde{k}$, that is how many triplets will have $\tilde{k}$ as the associated key value for the 112 key bit guessed in the procedure. Analogously to what we explained in Sect. 4.2, this is to say that for any possible master key value *k*, we can consider a statistic $\Sigma _k$ given by these counters, and test the ones with the higher counter as candidates (by eventually guessing the remaining 16 bits) by encrypting a plaintext under the candidate key and testing whether the ciphertext matches the one provided by the oracle.

In particular, if we test the candidates whose counter pass a certain threshold $\theta$, we have to test $2^{128}\beta$ key candidates, where $\beta$ is the false flag probability that indicates the probability of a random key candidate passing the fixed threshold. As we have seen, this $\beta$ depends on the values of *N*, $\theta$, which also determine the probability that the entire routine succedes, $\alpha$. In order to have the same success probability of at least 90% ( that is $\alpha <0.1$) as in the original attack, we choose the number of pairs $N=2^{60.96}$ and $\theta =4$. Then we have that for this choice of parameters $\beta =2^{16.85}$ and the data complexity is $D=2\cdot N=2^{61.96}$, that we need $\mathcal {S}=2^{4.96}$ structures. Furthermore, the time complexity will be given by $T_1$ of Eq. (5) and $T_2=2^{128}\beta (1-2^{64})$, for a total

$$\begin{aligned} T_1+T_2= 2^{112.57}+2^{111.15}= 2^{113.03} \end{aligned}$$

26-round encryptions. This also requires a memory complexity of $2^{112}\beta =2^{95.15}$ 112-bit keys in order to store the key candidates that satisfy the threshold.

#### Improved memory complexity

Let us now describe how to perform an attack with minimal memory thanks to our improved key recovery techniques. In order to do so, we do not perform the guess by generating all possible triplets and store the subkey values that pass the threshold. Instead, for each possible *N* plaintext-ciphertext pair, we do the key guessing exactly as before, at each step generating a triplet that we analyse on the fly. That is, we do not store any triplet or key candidate, but simply do the key guess to generate, one by one, all the possible good triplets for the distinguisher according to our improved key-guessing. As soon as we generate a good triplet, to which a 112-bit subkey candidate is associated, we guess the remaining 16 bits of the master key that are not yet determined, and we then test the correctness of the full key. If the key is wrong, we generate the next triplet and do the same until the right key is recovered.

The time complexity is then given by the time it takes to generate all the possible good triplets, that amounts as before to

$$\begin{aligned} T_0 = N_1 \cdot 2^{-21}(2^{19}+2^{19}+2^{24.32}+ 2^{13.79}\cdot 2^{10.43})\frac{1}{26\cdot 16}= N_1 \cdot 2^{-4.40} \end{aligned}$$

26-round encryptions generating a total of $N_1 \cdot 2^{-21}\cdot 2^{13.79-4.36}=N_1 \cdot 2^{-10.97}$ triplets. For each of this triplet, we need to guess the undetermined 16 bits of the master key. Therefore, the total complexity is

$$\begin{aligned} T_1= T_0 + N_1\cdot 2^{-10.97} \cdot 2^{16} \approx N_1 \cdot 2^{5.03}= 2^{120} \end{aligned}$$

for the choice of data complexity equal to $D=2\cdot N=2^{59.96}$, which is half as in the original attack, that is $N_1= 2^{114.96}$ and $\alpha <0.1$ (*i.e.* the probability of success is higher than 90%). Notice that in this case the bottleneck of the memory is given simply by the size of the tables that are built in the process, *i.e.* $2^{24.32}$ entries of two plaintext and one key guess which are then the size of $2^{25.32}$ 128-bit words. Finally, we remark that this variant of the attack was made possible thanks to the fact that our improved sieving leads to a number of good triplets that allows the enumeration of the 16 key bits not involved in the key guessing, without getting too close to exhaustively searching $2^{128}$ key values.

## Conclusion

We have proposed two different trade-offs to improve the previous best known attacks on GIFT-64: a related-key differential attack on 26 rounds [13].

We propose one that improves the memory complexity by a factor of $2^{77}$ and a time complexity reduced by about $2^3$, while having the same data complexity. The other one allows to reduce the time complexity by a factor of $2^{10}$ and the memory by a factor $2^{7.5}$ at the cost of increasing the data by a factor 2. We have implemented our techniques in a toy cipher in order to verify their validity (described in Appendix E).

We also believe that the ideas we applied, that are very generic, should be taken into account when building key-recovery attacks with differential cryptanalysis. We believe similar improvements would also apply in the case of the best single-key attacks on GIFT-64, on 21 rounds, but as it is a multiple-differential attack, the techniques would need some adaptation, and we leave this case as an interesting open problem. The final aim of our techniques is to algorithmically optimize the cost of the different steps of the key recovery part, by applying a parallel search on efficient subsets and an efficient merging of the partial solutions.

Finally, we would like to point out that improving the needs of memory complexity is a task that should be considered more often.

## Figures and Tables

**Fig. 3:**
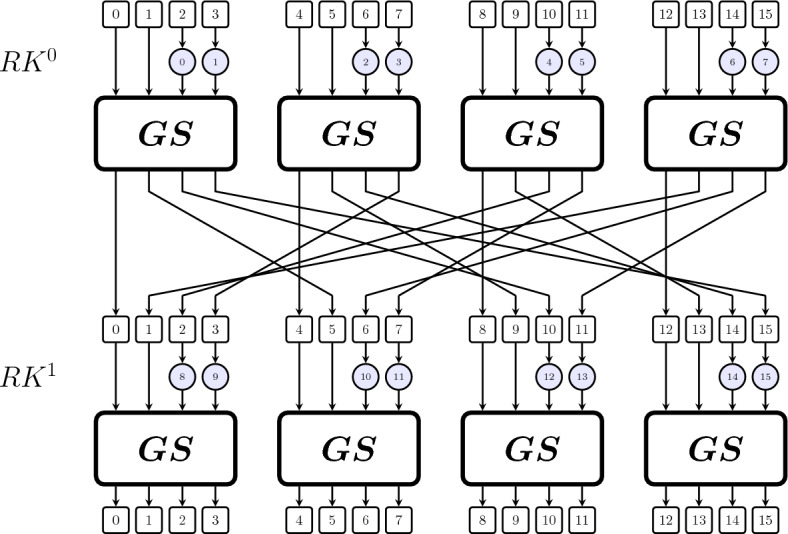
Two consecutive rounds of the GIFT-like toy cipher. Master key bits are represented in blue circles (Color figure online)

**Fig. 4:**
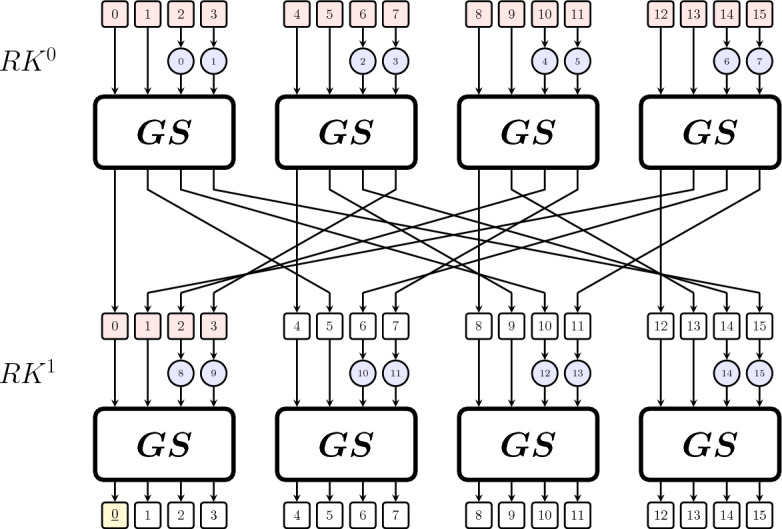
Key recovery with improved triplet sieving (early rounds). Active bits with variable difference are depicted in red squares, while active bits with difference set to 1 are underlined and depicted in yellow squares. White squares represent inactive bits. Master key bits are represented in blue circles (Color figure online)

**Fig. 5:**
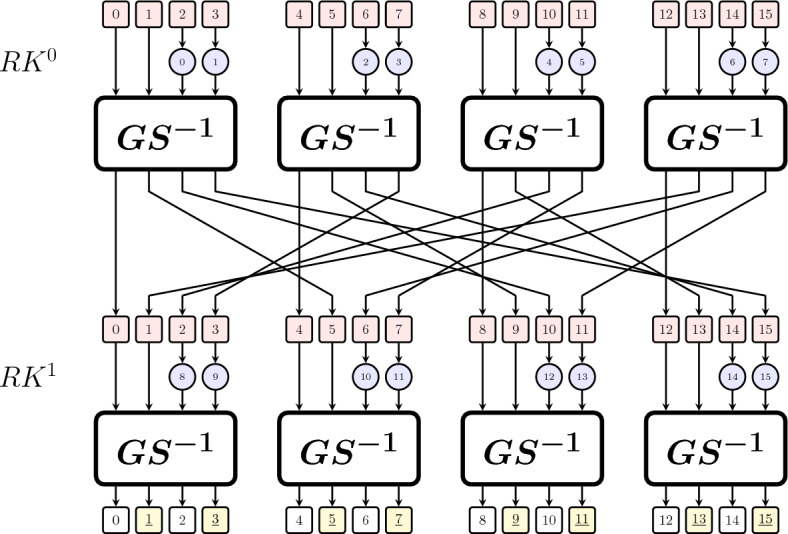
Key recovery for with parallel matching (final rounds). Active bits with variable difference are underlined and depicted in red squares, while active bits with difference set to 1 are depicted in yellow squares. White squares represent inactive bits. Master key bits are represented in blue circles (Color figure online)

## A Tree-guessing for $y_0, y_1, y_2, y_3$

For $y_0$, we use the following guessing strategy:

- if $$(x_1,x_0)=(0,0)$$, then $$y_0=x_3 \oplus k_1$$
- if $$(x_1,x_0)=(0,1)$$, then $$y_0=x_3 \oplus x_2\oplus k_1\oplus k_0$$
- if $$(x_1,x_0)=(1,0)$$, then $$y_0=x_3 \oplus k_1$$
- if $$(x_1,x_0)=(1,1)$$, then $$y_0=x_2 \oplus k_0$$

For $y_1$, we use the following guessing strategy:

- if $$(x_1,x_0)=(0,0)$$, then $$y_0=x_2 \oplus k_0 \oplus x_1$$
- if $$(x_1,x_0)=(0,1)$$, then $$y_0=x_3 \oplus k_1\oplus x_1$$
- if $$(x_1,x_0)=(1,0)$$, then $$y_0=x_2 \oplus k_0 \oplus x_1$$
- if $$(x_1,x_0)=(1,1)$$, then $$y_0=x_3 \oplus x_2\oplus k_1\oplus k_0\oplus x_1$$

For $y_2$, we use the following guessing strategy:

- Guess $$ k_0$$
  - if $$(x_1,x_2\oplus k_0)=(0,0)$$, then $$y_2=x_0+x_3+k_1$$;
  - if $$(x_1,x_2\oplus k_0)=(0,1)$$, then $$y_2= x_0$$;
  - if $$(x_1,x_2\oplus k_0)=(1,0)$$, then $$y_2= x_0+1$$;
  - if $$(x_1,x_2\oplus k_0)=(1,1)$$, then $$y_0=x_0 + x_3+k_1$$.

For $y_3$, we use the following guessing strategy:

- Guess $$ k_0$$
  - if $$x_2\oplus k_0=0$$, then $$y_3=x_3\oplus k_1 \oplus x_1 \oplus x_0 \oplus 1$$;
  - if $$x_2\oplus k_0=1$$, then $$y_3=x_1\oplus x_0$$

## B Tables for the triplet sieve of *RK*0 and *RK*1

See Tables 4, 5, 6, 7 and 8.

**Table 4:** Triplet sieving for Type 2 ($XXXX\rightarrow 1000$)

Input difference	$\nabla _{\delta \rightarrow \Delta }$	Pre sieve	# of possible keys	# of triplets
3	$\{ \texttt {5}, \texttt {6} \}$	$2^{-1}$	$2^{ 0}$	$2^{-1}$
7	$\{ \texttt {a}, \texttt {d} \}$	$2^{-1}$	$2^{ 0}$	$2^{-1}$
8	$\{ \texttt {4}, \texttt {c} \}$	$2^{-2}$	$2^{ 1}$	$2^{-1}$
9	$\{ \texttt {1}, \texttt {8} \}$	$2^{-1}$	$2^{ 0}$	$2^{-1}$
a	$\{ \texttt {3}, \texttt {9} \}$	$2^{-1}$	$2^{ 0}$	$2^{-1}$
c	$\{ \texttt {7}, \texttt {b} \}$	$2^{-2}$	$2^{ 1}$	$2^{-1}$
d	$\{ \texttt {2}, \texttt {f} \}$	$2^{-1}$	$2^{ 0}$	$2^{-1}$
e	$\{ \texttt {0}, \texttt {e} \}$	$2^{-1}$	$2^{ 0}$	$2^{-1}$

**Table 5:** Triplet sieving for Type 3 ($XXXX\rightarrow 0100$)

Input difference	$\nabla _{\delta \rightarrow \Delta }$	Pre sieve	# of possible keys	# of triplets
3	$\{ \texttt {0}, \texttt {1}, \texttt {2}, \texttt {3} \}$	$2^{ 0}$	$2^{ 0}$	$2^{ 0}$
7	$\{ \texttt {8}, \texttt {9}, \texttt {e}, \texttt {f} \}$	$2^{ 0}$	$2^{ 0}$	$2^{ 0}$
b	$\{ \texttt {6}, \texttt {7}, \texttt {c}, \texttt {d} \}$	$2^{ 0}$	$2^{ 0}$	$2^{ 0}$
f	$\{ \texttt {4}, \texttt {5}, \texttt {a}, \texttt {b} \}$	$2^{ 0}$	$2^{ 0}$	$2^{ 0}$

**Table 6:** Triplet sieving for Type 4 ($XXXX \rightarrow 00X0$). The triplet sieve takes into account the key absorption used for $\delta =0$

Input difference	$\nabla _{\delta \rightarrow \Delta }$	Pre sieve	# of possible keys	# of triplets
0	$\mathbb {F}_2^4$	$2^{0}$	$2^1$	$2^{1}$
3	$\{ \texttt {9}, \texttt {a}, \texttt {c}, \texttt {f} \}$	$2^{ 0}$	$2^{ 0}$	$2^{ 0}$
6	$\{ \texttt {0}, \texttt {2}, \texttt {4}, \texttt {6} \}$	$2^{-1}$	$2^{ 0}$	$2^{ -1}$
a	$\{ \texttt {1}, \texttt {b} \}$	$2^{-1}$	$2^{ 0}$	$2^{-1}$
b	$\{ \texttt {5}, \texttt {e} \}$	$2^{-1}$	$2^{ 0}$	$2^{-1}$
e	$\{ \texttt {3}, \texttt {d} \}$	$2^{-1}$	$2^{ 0}$	$2^{-1}$
f	$\{ \texttt {7}, \texttt {8} \}$	$2^{-1}$	$2^{ 0}$	$2^{-1}$

**Table 7:** Triplet sieving for Type 5 ($XXXX\rightarrow X000$). The triplet sieve takes into account the key absorption used for $\delta =0$

Input difference	$\nabla _{\delta \rightarrow \Delta }$	Pre sieve	# of possible keys	# of triplets
0	$\mathbb {F}_2^4$	$2^{0}$	$2^1$	$2^{1}$
3	$\{ \texttt {5}, \texttt {6} \}$	$2^{-1}$	$2^{ 0}$	$2^{-1}$
7	$\{ \texttt {a}, \texttt {d} \}$	$2^{-1}$	$2^{ 0}$	$2^{-1}$
8	$\{ \texttt {4}, \texttt {c} \}$	$2^{-2}$	$2^{ 1}$	$2^{-1}$
9	$\{ \texttt {1}, \texttt {8} \}$	$2^{-1}$	$2^{ 0}$	$2^{-1}$
a	$\{ \texttt {3}, \texttt {9} \}$	$2^{-1}$	$2^{ 0}$	$2^{-1}$
c	$\{ \texttt {7}, \texttt {b} \}$	$2^{-2}$	$2^{ 1}$	$2^{-1}$
d	$\{ \texttt {2}, \texttt {f} \}$	$2^{-1}$	$2^{ 0}$	$2^{-1}$
e	$\{ \texttt {0}, \texttt {e} \}$	$2^{-1}$	$2^{ 0}$	$2^{-1}$

**Table 8:** Triplet sieving for Type 6 ($X1XX\rightarrow 0010$). The triplet sieve takes into account the key absorption used for $\delta =0$

Input difference	$\nabla _{\delta \rightarrow \Delta }$	Pre sieve	# of possible keys	# of triplets
3	$\{ \texttt {9}, \texttt {a}, \texttt {c}, \texttt {f} \}$	$2^{ 0}$	$2^{ 0}$	$2^{ 0}$
6	$\{ \texttt {0}, \texttt {2}, \texttt {4}, \texttt {6} \}$	$2^{-1}$	$2^{ 0}$	$2^{ -1}$
a	$\{ \texttt {1}, \texttt {b} \}$	$2^{-1}$	$2^{ 0}$	$2^{-1}$
b	$\{ \texttt {5}, \texttt {e} \}$	$2^{-1}$	$2^{ 0}$	$2^{-1}$
e	$\{ \texttt {3}, \texttt {d} \}$	$2^{-1}$	$2^{ 0}$	$2^{-1}$
f	$\{ \texttt {7}, \texttt {8} \}$	$2^{-1}$	$2^{ 0}$	$2^{-1}$

## C Tables for pre-sieving of $X^{24}$

See Tables 9 and 10.

**Table 9:** Pre-sieving for $XXXX\rightarrow X0X0$. The average pre-sieve is $2^{-0.87}$; for each sieved triplet, a guess of $2^{0.87}$ is then needed

Input difference	$\nabla _{\delta \rightarrow \Delta }$	Pre-sieve	# of possible keys
0	$\mathbb {F}_2^4$	$2^0$	$2^2$
5	$\{ \texttt {8}, \texttt {b}, \texttt {d}, \texttt {e} \}$	$2^{ 0}$	$2^{ 0}$
6	$\{ \texttt {2}, \texttt {3}, \texttt {4}, \texttt {5}, \texttt {9}, \texttt {a}, \texttt {c}, \texttt {f} \}$	$2^{ 0}$	$2^{ 1}$
7	$\{ \texttt {0}, \texttt {1}, \texttt {6}, \texttt {7} \}$	$2^{ 0}$	$2^{ 0}$
8	$\{ \texttt {3}, \texttt {7}, \texttt {b}, \texttt {f} \}$	$2^{-2}$	$2^{ 2}$
9	$\{ \texttt {0}, \texttt {4}, \texttt {9}, \texttt {d} \}$	$2^{-1}$	$2^{ 1}$
a	$\{ \texttt {2}, \texttt {6}, \texttt {8}, \texttt {c} \}$	$2^{-1}$	$2^{ 1}$
b	$\{ \texttt {1}, \texttt {5}, \texttt {a}, \texttt {e} \}$	$2^{-1}$	$2^{ 1}$
c	$\{ \texttt {4}, \texttt {6}, \texttt {8}, \texttt {a} \}$	$2^{-1}$	$2^{ 1}$
d	$\{ \texttt {1}, \texttt {3}, \texttt {c}, \texttt {e} \}$	$2^{ 0}$	$2^{ 0}$
e	$\{ \texttt {5}, \texttt {7}, \texttt {9}, \texttt {b} \}$	$2^{-1}$	$2^{ 1}$
f	$\{ \texttt {0}, \texttt {2}, \texttt {d}, \texttt {f} \}$	$2^{ 0}$	$2^{ 0}$

**Table 10:** Pre-sieving for $XXXX\rightarrow 0X0X$. The average pre-sieve is $2^{-0.91}$; for each sieved triplet, a guess of $2^{0.91}$ is then needed

Input difference	$\nabla _{\delta \rightarrow \Delta }$	Pre-sieve	# of possible keys
0	$\mathbb {F}_2^4$	$2^0$	$2^2$
1	$\{ \texttt {2}, \texttt {3}, \texttt {a}, \texttt {b} \}$	$2^{-1}$	$2^{ 1}$
3	$\{ \texttt {5}, \texttt {6}, \texttt {d}, \texttt {e} \}$	$2^{-1}$	$2^{ 1}$
4	$\{ \texttt {0}, \texttt {4} \}$	$2^{-2}$	$2^{ 1}$
5	$\{ \texttt {9}, \texttt {c} \}$	$2^{-1}$	$2^{ 0}$
6	$\{ \texttt {1}, \texttt {7} \}$	$2^{-1}$	$2^{ 0}$
7	$\{ \texttt {8}, \texttt {f} \}$	$2^{-1}$	$2^{ 0}$
9	$\{ \texttt {6}, \texttt {7}, \texttt {e}, \texttt {f} \}$	$2^{-1}$	$2^{ 1}$
a	$\{ \texttt {0}, \texttt {3}, \texttt {5}, \texttt {7}, \texttt {9}, \texttt {a}, \texttt {d}, \texttt {f} \}$	$2^{ 0}$	$2^{ 1}$
b	$\{ \texttt {0}, \texttt {2}, \texttt {9}, \texttt {b} \}$	$2^{ 0}$	$2^{ 0}$
c	$\{ \texttt {1}, \texttt {d} \}$	$2^{-2}$	$2^{ 1}$
d	$\{ \texttt {5}, \texttt {8} \}$	$2^{-1}$	$2^{ 0}$
e	$\{ \texttt {2}, \texttt {4}, \texttt {6}, \texttt {8}, \texttt {a}, \texttt {c} \}$	$2^{-1}$	$2^{ 1}$
f	$\{ \texttt {1}, \texttt {3}, \texttt {4}, \texttt {b}, \texttt {c}, \texttt {e} \}$	$2^{ 0}$	$2^{ 0}$

## D Tables for guessing $RK^{23}$ and $RK^{22}$

See Tables 11, 12, 13, 14, 15 and 16.

**Table 11:** Pre-sieving for $XXXX\rightarrow 00XX$. The average pre-sieve is $2^{-0.68}$. The average number of triplets generated is $2^{0.68}$

Input difference	$\nabla _{\delta \rightarrow \Delta }$	Pre-sieve	# of possible keys	# of triplets
0	$\mathbb {F}_2^4$	$2^0$	$2^2$	$2^2$
1	$\{ \texttt {0}, \texttt {1}, \texttt {2}, \texttt {3} \}$	$2^{ 0}$	$2^{ 0}$	$2^{ 0}$
3	$\{ \texttt {c}, \texttt {d}, \texttt {e}, \texttt {f} \}$	$2^{ 0}$	$2^{ 0}$	$2^{ 0}$
5	$\{ \texttt {9}, \texttt {b}, \texttt {c}, \texttt {e} \}$	$2^{ 0}$	$2^{ 0}$	$2^{ 0}$
6	$\{ \texttt {1}, \texttt {3}, \texttt {5}, \texttt {7}, \texttt {9}, \texttt {b}, \texttt {d}, \texttt {f} \}$	$2^{-1}$	$2^{ 2}$	$2^{ 1}$
7	$\{ \texttt {0}, \texttt {2}, \texttt {5}, \texttt {7} \}$	$2^{ 0}$	$2^{ 0}$	$2^{ 0}$
9	$\{ \texttt {4}, \texttt {6}, \texttt {d}, \texttt {f} \}$	$2^{ 0}$	$2^{ 0}$	$2^{ 0}$
a	$\{ \texttt {0}, \texttt {2}, \texttt {4}, \texttt {6}, \texttt {8}, \texttt {a}, \texttt {c}, \texttt {e} \}$	$2^{-1}$	$2^{ 2}$	$2^{ 1}$
b	$\{ \texttt {1}, \texttt {3}, \texttt {8}, \texttt {a} \}$	$2^{ 0}$	$2^{ 0}$	$2^{ 0}$
d	$\{ \texttt {5}, \texttt {7}, \texttt {8}, \texttt {a} \}$	$2^{ 0}$	$2^{ 0}$	$2^{ 0}$
f	$\{ \texttt {4}, \texttt {6}, \texttt {9}, \texttt {b} \}$	$2^{ 0}$	$2^{ 0}$	$2^{ 0}$

**Table 12:** Pre-sieving for $XXXX\rightarrow 00X1$. The average pre-sieve is $2^{-1.19}$; for each sieved triplet, a guess of $2^{0.19}$ is then needed

Input difference	$\nabla _{\delta \rightarrow \Delta }$	Pre-sieve	# of possible keys	# of triplets
1	$\{ \texttt {0}, \texttt {1}, \texttt {2}, \texttt {3} \}$	$2^{ 0}$	$2^{ 0}$	$2^{ 0}$
3	$\{ \texttt {c}, \texttt {d}, \texttt {e}, \texttt {f} \}$	$2^{ 0}$	$2^{ 0}$	$2^{ 0}$
5	$\{ \texttt {9}, \texttt {c} \}$	$2^{-1}$	$2^{ 0}$	$2^{-1}$
6	$\{ \texttt {1}, \texttt {7}, \texttt {b}, \texttt {d} \}$	$2^{-1}$	$2^{ 1}$	$2^{ 0}$
7	$\{ \texttt {2}, \texttt {5} \}$	$2^{-1}$	$2^{ 0}$	$2^{-1}$
9	$\{ \texttt {6}, \texttt {f} \}$	$2^{-1}$	$2^{ 0}$	$2^{-1}$
a	$\{ \texttt {0}, \texttt {4}, \texttt {a}, \texttt {e} \}$	$2^{-1}$	$2^{ 1}$	$2^{ 0}$
b	$\{ \texttt {3}, \texttt {8} \}$	$2^{-1}$	$2^{ 0}$	$2^{-1}$
d	$\{ \texttt {5}, \texttt {7}, \texttt {8}, \texttt {a} \}$	$2^{ 0}$	$2^{ 0}$	$2^{ 0}$
f	$\{ \texttt {4}, \texttt {6}, \texttt {9}, \texttt {b} \}$	$2^{ 0}$	$2^{ 0}$	$2^{ 0}$

**Table 13:** Pre-sieving for $XXXX\rightarrow 0XX0$. The average pre-sieve is $2^{-1}$; for each sieved triplet, a guess of $2^{1}$ is then needed

Input difference	$\nabla _{\delta \rightarrow \Delta }$	Pre-sieve	# of possible keys	# of triplets
0	$\mathbb {F}_2^4$	$2^0$	$2^2$	$2^2$
4	$\{ \texttt {2}, \texttt {6}, \texttt {8}, \texttt {c} \}$	$2^{-1}$	$2^{ 1}$	$2^{ 0}$
5	$\{ \texttt {1}, \texttt {4}, \texttt {b}, \texttt {e} \}$	$2^{ 0}$	$2^{ 0}$	$2^{ 0}$
6	$\{ \texttt {3}, \texttt {5}, \texttt {9}, \texttt {f} \}$	$2^{-1}$	$2^{ 1}$	$2^{ 0}$
7	$\{ \texttt {0}, \texttt {7}, \texttt {a}, \texttt {d} \}$	$2^{ 0}$	$2^{ 0}$	$2^{ 0}$
9	$\{ \texttt {4}, \texttt {7}, \texttt {d}, \texttt {e} \}$	$2^{ 0}$	$2^{ 0}$	$2^{ 0}$
a	$\{ \texttt {2}, \texttt {3}, \texttt {5}, \texttt {6}, \texttt {8}, \texttt {9}, \texttt {c}, \texttt {f} \}$	$2^{ 0}$	$2^{ 1}$	$2^{ 1}$
b	$\{ \texttt {0}, \texttt {1}, \texttt {a}, \texttt {b} \}$	$2^{ 0}$	$2^{ 0}$	$2^{ 0}$
c	$\{ \texttt {1}, \texttt {3}, \texttt {5}, \texttt {7}, \texttt {9}, \texttt {b}, \texttt {d}, \texttt {f} \}$	$2^{-1}$	$2^{ 2}$	$2^{ 1}$
e	$\{ \texttt {0}, \texttt {2}, \texttt {4}, \texttt {6}, \texttt {8}, \texttt {a}, \texttt {c}, \texttt {e} \}$	$2^{-1}$	$2^{ 2}$	$2^{ 1}$

**Table 14:** Triplets sieving for $XXXX\rightarrow XX00$. The average pre-sieve is $2^{-0.83}$; for each sieved triplet, a guess of $2^{0.83}$ is then needed

Input difference	$\nabla _{\delta \rightarrow \Delta }$	Pre-sieve	# of possible keys	# of triplets
0	$\mathbb {F}_2^4$	$2^0$	$2^2$	$2^2$
1	$\{ \texttt {c}, \texttt {d} \}$	$2^{-1}$	$2^{ 0}$	$2^{-1}$
2	$\{ \texttt {4}, \texttt {6}, \texttt {8}, \texttt {a} \}$	$2^{-1}$	$2^{ 1}$	$2^{ 0}$
3	$\{ \texttt {1}, \texttt {2} \}$	$2^{-1}$	$2^{ 0}$	$2^{-1}$
4	$\{ \texttt {3}, \texttt {7}, \texttt {b}, \texttt {f} \}$	$2^{-2}$	$2^{ 2}$	$2^{ 0}$
5	$\{ \texttt {0}, \texttt {5} \}$	$2^{-1}$	$2^{ 0}$	$2^{-1}$
7	$\{ \texttt {9}, \texttt {e} \}$	$2^{-1}$	$2^{ 0}$	$2^{-1}$
9	$\{ \texttt {7}, \texttt {e} \}$	$2^{-1}$	$2^{ 0}$	$2^{-1}$
a	$\{ \texttt {3}, \texttt {5}, \texttt {9}, \texttt {f} \}$	$2^{-1}$	$2^{ 1}$	$2^{ 0}$
b	$\{ \texttt {0}, \texttt {b} \}$	$2^{-1}$	$2^{ 0}$	$2^{-1}$
c	$\{ \texttt {1}, \texttt {4}, \texttt {6}, \texttt {8}, \texttt {a}, \texttt {d}\}$	$2^{ 0}$	3	3
d	$\{ \texttt {1}, \texttt {3}, \texttt {c}, \texttt {e} \}$	$2^{ 0}$	$2^{ 0}$	$2^{ 0}$
e	$\{ \texttt {2}, \texttt {4}, \texttt {5}, \texttt {6}, \texttt {7}, \texttt {8}, \texttt {9}, \texttt {a}, \texttt {b}, \texttt {c} \}$	$2^{ 0}$	$2^{ 1}$	$2^{ 1}$
f	$\{ \texttt {0}, \texttt {2}, \texttt {d}, \texttt {f} \}$	$2^{ 0}$	$2^{ 0}$	$2^{ 0}$

**Table 15:** Triplet sieving for $XXXX\rightarrow X00X$. The average pre-sieve is $2^{-0.71}$; for each sieved triplet, a guess of $2^{0.71}$ is then needed

Input difference	$\nabla _{\delta \rightarrow \Delta }$	Pre-sieve	# of possible keys	# of triplets
0	$\mathbb {F}_2^4$	$2^0$	$2^2$	$2^2$
1	$\{ \texttt {2}, \texttt {3}, \texttt {4}, \texttt {5} \}$	$2^{ 0}$	$2^{ 0}$	$2^{ 0}$
3	$\{ \texttt {8}, \texttt {b}, \texttt {d}, \texttt {e} \}$	$2^{ 0}$	$2^{ 0}$	$2^{ 0}$
5	$\{ \texttt {9}, \texttt {a}, \texttt {c}, \texttt {f} \}$	$2^{ 0}$	$2^{ 0}$	$2^{ 0}$
6	$\{ \texttt {0}, \texttt {1}, \texttt {6}, \texttt {7} \}$	$2^{ 0}$	$2^{ 0}$	$2^{ 0}$
8	$\{ \texttt {1}, \texttt {9} \}$	$2^{-2}$	$2^{ 1}$	$2^{-1}$
9	$\{ \texttt {6}, \texttt {f} \}$	$2^{-1}$	$2^{ 0}$	$2^{-1}$
a	$\{ \texttt {0}, \texttt {a} \}$	$2^{-1}$	$2^{ 0}$	$2^{-1}$
b	$\{ \texttt {7}, \texttt {c} \}$	$2^{-1}$	$2^{ 0}$	$2^{-1}$
c	$\{ \texttt {2}, \texttt {4}, \texttt {6}, \texttt {8}, \texttt {a}, \texttt {e} \}$	$2^{-1}$	$2^{ 1}$	$2^{ 0}$
d	$\{ \texttt {1}, \texttt {3}, \texttt {5}, \texttt {8}, \texttt {c}, \texttt {e} \}$	$2^{ 0}$	$2^{ 0}$	$2^{ 0}$
e	$\{ \texttt {3}, \texttt {5}, \texttt {7}, \texttt {9}, \texttt {b}, \texttt {d} \}$	$2^{-1}$	$2^{ 1}$	$2^{ 0}$
f	$\{ \texttt {0}, \texttt {2}, \texttt {4}, \texttt {b}, \texttt {d}, \texttt {f} \}$	$2^{ 0}$	$2^{ 0}$	$2^{ 0}$

**Table 16:** Triplet sieving for $XXXX\rightarrow X001$. The average pre-sieve is $2^{-1.09}$; for each sieved triplet, a guess of $2^{0.09}$ is then needed

Input difference	$\nabla _{\delta \rightarrow \Delta }$	Pre-sieve	# of possible keys	# of triplets
1	$\{ \texttt {2}, \texttt {3}, \texttt {4}, \texttt {5} \}$	$2^{ 0}$	$2^{ 0}$	$2^{ 0}$
3	$\{ \texttt {8}, \texttt {b}, \texttt {d}, \texttt {e} \}$	$2^{ 0}$	$2^{ 0}$	$2^{ 0}$
5	$\{ \texttt {9}, \texttt {a}, \texttt {c}, \texttt {f} \}$	$2^{ 0}$	$2^{ 0}$	$2^{ 0}$
6	$\{ \texttt {0}, \texttt {1}, \texttt {6}, \texttt {7} \}$	$2^{ 0}$	$2^{ 0}$	$2^{ 0}$
8	$\{ \texttt {1}, \texttt {9} \}$	$2^{-2}$	$2^{ 1}$	$2^{-1}$
9	$\{ \texttt {6}, \texttt {f} \}$	$2^{-1}$	$2^{ 0}$	$2^{-1}$
a	$\{ \texttt {0}, \texttt {a} \}$	$2^{-1}$	$2^{ 0}$	$2^{-1}$
b	$\{ \texttt {7}, \texttt {c} \}$	$2^{-1}$	$2^{ 0}$	$2^{-1}$
c	$\{ \texttt {2}, \texttt {e} \}$	$2^{-2}$	$2^{ 1}$	$2^{-1}$
d	$\{ \texttt {5}, \texttt {8} \}$	$2^{-1}$	$2^{ 0}$	$2^{-1}$
e	$\{ \texttt {3}, \texttt {d} \}$	$2^{-1}$	$2^{ 0}$	$2^{-1}$
f	$\{ \texttt {4}, \texttt {b} \}$	$2^{-1}$	$2^{ 0}$	$2^{-1}$

## E Experimental verification

In order to verify the correctness of the improved attack we propose on GIFT, and because the whole attack is not practical, we have implemented the new introduced techniques and ideas when applied on a toy-cipher based on GIFT.

First, we describe our improved upper rounds guessing part, applied on a key-recovery attack on a GIFT-like toy cipher with 4 Sboxes per round. We have been able to experimentally verify the predicted complexities using the techniques we propose in the paper. For the sake of simplicity, so that we can better verify our new proposed techniques, we have not included the absorption technique that was already introduced in [5].

We have also verified that the amount of undetermined key material for the triplets thus generated is the same as our analysis expects. This confirms that the estimate for the size of the lists that are used for merging in Sect. 5.3 are indeed accurate.

Finally, we verify that the sieving probability predicted by our techniques in Sect. 5.3 is correct. This, together with the confirmation of the correctness of the expected size of the lists used for merging, and the fact that the complexity of the parallel matching algorithm was already experimentally verified in [6], confirms that the techniques used in Sect. 5.3 for an efficient guess of $RK_{22}$ and $RK_{21}$ yield the correct complexities. In particular, we show the impact that guessing in groups and then using efficient merging algorithms like parallel matching [12] can have.

In order to have a clear confirmation of the correctness of the respective techniques, we have chosen to implement both of them separately, as there is no reason to expect that combining both of them in the same attack would alter the results.

### E.1 Description of the toy-cipher

A round of the GIFT-like toy-cipher we have considered is composed of AddRK, SboxLayer, PermuteBits. A state is divided into four words of 4-bit each, so $n=16$. The master key *mk* is $KL=16$ bits long. A graphical representation of two consecutive rounds is provided in Fig. 3.

In particular, AddRK is a XOR of the bits

$$\begin{aligned} rk_0=MK[2\cdot (4r+i) \pmod {KL}] \end{aligned}$$

and

$$\begin{aligned} rk_1=MK[2\cdot (4r+i)+1\pmod {KL}] \end{aligned}$$

to the two MSB ($x_2$ and $x_3$ resp.) of the *i*-th word in round *r*, where *MK*[*j*] is the *j*-th bit of the masterkey. SboxLayer is the parallel application of the GIFT Sbox to each nibble. Finally, PermuteBits permutes bits according to the following $\pi$:

$$\begin{aligned} \pi (i)= 4\cdot i \pmod {16} + i \pmod {16}. \end{aligned}$$

### E.2 Key recovery with improved triplet sieving

We want to perform two rounds of key recovery considering a distinguisher whose input difference is $\texttt {1000 0000 0000 0000}$, as shown in Fig. 4. We here focus on estimating the improved filtering as of Sect. 5.2, as well as the frequency with which some bits may or may not be determined due to this (which is the underlying assumption for the cost estimates of the final guess in Sect. 5.3), both experimentally verified. We recall that for the sake of clarity, we will not make use of the key absorption technique, since it was already introduced in [5].

We now estimate the number of triplets generated as done in Sect. 5.2.

#### Sbox 1, round 1

For the transition $XXXX\rightarrow X000$, we consider first the case when the input difference $\delta = \texttt {0}$ (which happens with probability $2^{-4}$) and we want to determine the output bit $y_0$. This needs 1 bit guess instead of 2 thanks to trees. Next, when $\delta \ne \texttt {0}$, the output difference must be $\Delta =\texttt {1}$. In this case, our technique generates $2^{-1}$ triplets for each of the possible eight input differences (as can be seen in Table 4). This implies that each pair generates on average

$$\begin{aligned} \frac{1}{2^{4}} \cdot 2 + 8 \cdot \frac{1}{2^{4}} \cdot 2^{-1}=\frac{6}{2^4} \end{aligned}$$

instead of the naive $2^{-1}$. Furthermore, for the $\frac{2}{2^4}$ generated triplets with input difference zero, due to the tree of $y_0$, half of them *only* have $rk_0$ determined, one fourth *only* $rk_1$ and one fourth only $rk_0\oplus rk_1$. In other words, out of the $\frac{6}{2^4}$ triplets generated in total, $\frac{1}{3}$ of them will have one bit of *MK*[0, 1] undetermined. In particular, $\frac{1}{6}$ have *MK*[0] undetermined, for $\frac{1}{12}$ triplets have *MK*[1] undetermined, for $\frac{1}{12}$ triplets *MK*[0] and *MK*[1] are undetermined, but $MK[0]\oplus MK[1]$ is determined.

#### Sbox 2, round 1

Similarly, for the transition $XXXX\rightarrow 000X$, when the input difference $\delta = \texttt {0}$, we can determine the output bit $y_3$ with 3 instead of 4 guesses. When $\Delta =\texttt {8}$, our technique generates on average $2^{-1}$ triplets for four of the six input differences and 1 for the other two (Table 2). That means that each pair generates

$$\begin{aligned} \frac{1}{2^{4}} \cdot 3 + 4 \cdot \frac{1}{2^{4}} \cdot 2^{-1}+ 2\cdot \frac{1}{2^{4}} \cdot 2 =\frac{7}{2^4} \end{aligned}$$

instead of the naive $2^{-1}$. As before, we observe that for the $\frac{3}{2^4}$ triplets generated with input difference $\texttt {0}$, for half of them $rk_1$ is undetermined, and therefore bit *MK*[3] is undetermined for $\frac{1}{7}$ of all generated triplets on average.

#### Sbox 3, round 1

For $XXXX \rightarrow 00X0$, when the input difference $\delta = \texttt {0}$, we can determine the output bit $y_2$ with 3 instead of 4 guesses. When $\Delta =\texttt {8}$, our techniques generate on average $2^{-1}$ triplets for four of the six input differences and 1 for the other two (Table 6). That means that each triplet generates

$$\begin{aligned} \frac{1}{2^{4}} \cdot 3 + 4 \cdot \frac{1}{2^{4}} \cdot 2^{-1}+ 2\cdot \frac{1}{2^{4}} \cdot 2 =\frac{7}{2^4} \end{aligned}$$

on average. As with Sbox 1, since the tree of $y_2$ is similar to the one of $y_3$, we expect $\frac{1}{7}$ of all generated triplets to have bit *MK*[4] undetermined.

#### Sbox 4, round 1

For $XXXX \rightarrow 0X00$, we can determine the output bit $y_1$ with 2 (instead of 4) guesses when $\delta =0$ (therefore $\Delta =0$). Otherwise, for $\delta \ne 0$, 1 triplet is generated for each of the four possible input differences. In total

$$\begin{aligned} \frac{1}{2^{4}} \cdot 2 + 4 \cdot \frac{1}{2^{4}} \cdot 1=\frac{6}{2^4} \end{aligned}$$

triplets are generated on average. Given the tree for $y_1$, we expect $\frac{1}{3}$ of all generated triplets to have one bit of *MK*[6, 7] undetermined. In particular, $\frac{1}{6}$ of the triplets have *MK*[7] undetermined, $\frac{1}{12}$ have undetermined *MK*[6], and the remaining $\frac{1}{12}$ have *MK*[6] and *MK*[7] undetermined, but $MK[6]\oplus MK[7]$ is determined (Table 17).

**Table 17:** Triplet sieving for $XXXX\rightarrow 0X00$ *without* key absorption

Input difference	$\nabla _{\delta \rightarrow \Delta }$	Pre-sieve	# of possible keys	# of triplets
0	$\mathbb {F}_2^2$	$2^{0}$	$2^{1}$	$2^{1}$
3	$\{ \texttt {0}, \texttt {1}, \texttt {2}, \texttt {3} \}$	$2^{ 0}$	$2^{ 0}$	$2^{ 0}$
7	$\{ \texttt {8}, \texttt {9}, \texttt {e}, \texttt {f} \}$	$2^{ 0}$	$2^{ 0}$	$2^{ 0}$
b	$\{ \texttt {6}, \texttt {7}, \texttt {c}, \texttt {d} \}$	$2^{ 0}$	$2^{ 0}$	$2^{ 0}$
f	$\{ \texttt {4}, \texttt {5}, \texttt {a}, \texttt {b} \}$	$2^{ 0}$	$2^{ 0}$	$2^{ 0}$

#### Sbox 1, round 2

As with Sbox 0 of round 0, the transition $XXXX\rightarrow 1000$ generates $2^{-1}$ triplets for each of the possible eight input differences (Table 7), that is each pair generates on average

$$\begin{aligned} 8 \cdot \frac{1}{2^{4}} \cdot 2^{-1}=\frac{4}{2^{4}}. \end{aligned}$$

In this case, since no tree (or key absorption) was used, the masterkey bits are always determined.

#### Verification of the estimated number of triplets generated

From the analysis above, we estimate that for *N* random pairs (with *N* sufficiently large)

$$\begin{aligned} N\cdot \left( \frac{6}{2^4}\right) ^2\cdot \left( \frac{7}{2^4}\right) ^2 \cdot \frac{4}{2^{4}}= N\cdot \frac{7056}{2^{20}}\sim N\cdot 2^{-7.21} \end{aligned}$$

triplets should be generated, instead of the naive $N\cdot 2^{10} \cdot 2^{-16}=N\cdot 2^{-6}$. This is an improvement over the naive filter of $2^{-1.21}$ over a 10-bit guess. Indeed, when using $N=10\cdot 2^{20}$ random pairs, our experiment generates $71611\sim 2^{16.12}$ triplets, which is very close to the theoretically expected number of $70560\sim 2^{16.11}$ triplets.

#### Verification of the undetermined keybits

As stated above, we expect that

- *MK*[0] is determined for $1-\frac{1}{6}-\frac{1}{12}=\frac{3}{4}$ of the triplets (experimentally, 51517 out of 71611)
- *MK*[1] is determined for $1-\frac{1}{12}-\frac{1}{12}=\frac{5}{6}$ of the triplets (experimentally, 58273 out of 71611)
- $MK[0]\oplus MK[1]$ (but not *MK*[0, 1]) is determined $\frac{1}{12}$ of the triplets (experimentally, 6650 out of 71611)
- *MK*[2] is determined for $\frac{6}{7}$ of the triplets (experimentally, 62552 out of 71611)
- *MK*[4] is determined for $\frac{6}{7}$ of the triplets (experimentally, 61483 out of 71611)
- *MK*[6] is determined for $1-\frac{1}{12}-\frac{1}{12}=\frac{5}{6}$ of the triplets (experimentally, 58253 out of 71611)
- *MK*[7] is determined for $1-\frac{1}{6}-\frac{1}{12}=\frac{3}{4}$ of the triplets (experimentally, 51735 out of 71611)
- $MK[6]\oplus MK[7]$ (but not *MK*[6, 7]) is determined $\frac{1}{12}$ of the triplets (experimentally, 6684 out of 71611)
- *MK*[3, 6, 8, 9] is determined for all triplets (experimentally, 71611 out of 71611).

These results seem to follow quite closely our predictions. The highest discrepancy between theoretical and experimental results is given by the frequency with which *MK*[0] is determined (the observed frequency is $\sim 0.72$ instead of 0.75), which can be explained by the choice of *N* being not sufficiently large, and the existence of some dependencies that we do not consider in our theoretical analysis. However, due to the mismatch between theory and experiments varying only between 1%-4% for a relatively small sample size, we believe that these results show that the independency assumption used to carry out the analyis is convenient and yields reasonably accurate results, confirming in particular that the prediction of the amount of undetermined keybits (that is, the size of the lists that need to be merged) computed in Sect. 5.3 are indeed quite accurate.

### E.3 Key recovery using pre-sieving and parallel-matching

We also verified experimentally the significance of pre-sieving, thus confirming the relevance of the technique, and the importance of combining it with an efficient guessing method, such as separating the key material in groups and use algorithms like the parallel matching for efficient merging.

In order to verify this step and preserve a parallelism with the original attack we will consider a key guessing on the final rounds. This means doing a key recovery for a differential distinguisher whose output difference is $\texttt {aaaa}$, as represented in Fig. 5. A naive guessing procedure of the last rounds keys could be the following:

1. guess 8 bits of masterkey from round 1, for a complexity of $N \cdot 2^8$ Sbox evaluations, generating $N\cdot 2^{8}$ triplets.
2. for each of the $N\cdot 2^8$ triplets, guess the remaining 8 master keybits, for a complexity of $N \cdot 2^{16}$ Sbox evaluations.

The time complexity is dominated by step 2, amounting to circa $N \cdot 2^{16}$ Sbox evaluations (this complexity can be lowered guessing each Sbox singularly, but the bottleneck would still be this step).

However, we can see from Table 18 that for the transition $XXXX\rightarrow 0101$ it is possible to sieve $2^{-2.68}$ triplets taking into account the input difference of the pair, and the value of the two least significant bits. A better technique would be then

1. guess 8 bits of masterkey from round 1, for a complexity of $N \cdot 2^8$ Sbox evaluations and producing $N\cdot 2^{8}$ triplets. For each of the generated triplet, sieve according to their input difference and least significant bits. $N\cdot 2^{8-2.68\cdot 4}= N\cdot 2^{-2.72}$ triplets remain. This was experimentally verified with $2^{20}$ pairs, that generate $160952\sim 2^{17.3}$ triplets.
2. for each of the remaining triplets, guess the remaining 8 master keybits, for a complexity of $N \cdot 2^{-2.72+8}=N \cdot 2^{5.28}$ Sboxes.

The guessing complexity is now dominated by step 1, for a total of $N\cdot (2^8+2^{5.28})=N\cdot 2^{8.2}$ Sbox computations. We can further improve this complexity with a more efficient step 1 thanks to parallel matching. In fact, we can guess the first roundkey by dividing it into two groups (the first being Sbox 0 and Sbox 2, and the other Sbox 1 and Sbox 3) and making use of the parallel matching algorithm to filter out wrong triplets efficiently. In this case, in fact, the complexity of the merging using groups of $t_1$ and $t_2$ Sboxes, would then amount to

$$\begin{aligned}&N\cdot (2^{3\cdot t_1+4-2.68\cdot t_1}+2^{3\cdot t_2-2.68\cdot t_2}+2^{8-2.68\cdot (t_1+t_2)})= \\&\quad N\cdot (2^{0.32\cdot t_1}+2^{0.32\cdot t_1}+2^{8-2.68\cdot (t_1+t_2)}) \end{aligned}$$

which is $2^{1.71}$ table lookups for $t_1=t_2=2$, which we can compare to an Sbox computation since such tables are smaller than an Sbox. The bottleneck of this step would then be building the two lists to merge, that is $N\cdot 2^4$ Sbox computations, thus making the overall complexity dominated by step 2, for a total of $N\cdot 2^4+ N\cdot 2^{5.28}=N\cdot 2^{5.78}$.

**Table 18:** Pre-sieving for $XXXX\rightarrow 0101$. The average pre-sieve is $2^{-2.68}$

Input difference	$\nabla _{\delta \rightarrow \Delta }$	Pre-sieve
2	$\{ \texttt {8}, \texttt {a}, \texttt {c}, \texttt {e} \}$	$2^{-1}$
4	$\{ \texttt {0}, \texttt {2}, \texttt {4}, \texttt {6} \}$	$2^{-1}$
8	$\{ \texttt {3}, \texttt {b} \}$	$2^{-2}$
a	$\{ \texttt {5}, \texttt {f} \}$	$2^{-1}$
c	$\{ \texttt {1}, \texttt {d} \}$	$2^{-2}$
e	$\{ \texttt {7}, \texttt {9} \}$	$2^{-1}$

## Footnotes

1 The code relative to the experiments can be found here: https://github.com/f-canale/GIFT.

2 When using this improved sieving in intermediate rounds, we assume that the possible difference of a triplet is uniformly random (*i.e.* $\text {Pr}_{x,x'}(x\oplus x'=\delta )$ is the same for every input differences $\delta$). In other words, each input difference has the same amount of triplets that satisfy it.
